# A Coarse-Grained Model of Affinity Maturation Indicates the Importance of B-Cell Receptor Avidity in Epitope Subdominance

**DOI:** 10.3389/fimmu.2022.816634

**Published:** 2022-03-18

**Authors:** Victor Ovchinnikov, Martin Karplus

**Affiliations:** ^1^ Department of Chemistry and Chemical Biology, Harvard University, Cambridge, MA, United States; ^2^ Laboratoire de Chimie Biophysique, ISIS, Université de Strasbourg, Strasbourg, France

**Keywords:** germinal center, simulation, influenza, hemagglutinin, vaccination

## Abstract

The elicitation of broadly neutralizing antibodies (bnAbs) is a major goal in the design of vaccines against rapidly-mutating viruses. In the case of influenza, many bnAbs that target conserved epitopes on the stem of the hemagglutinin protein (HA) have been discovered. However, these antibodies are rare, are not boosted well upon reinfection, and often have low neutralization potency, compared to strain-specific antibodies directed to the HA head. Different hypotheses have been proposed to explain this phenomenon. We use a coarse-grained computational model of the germinal center reaction to investigate how B-cell receptor binding valency affects the growth and affinity maturation of competing B-cells. We find that receptors that are unable to bind antigen bivalently, and also those that do not bind antigen cooperatively, have significantly slower rates of growth, memory B-cell production, and, under certain conditions, rates of affinity maturation. The corresponding B-cells are predicted to be outcompeted by B-cells that bind bivalently and cooperatively. We use the model to explore strategies for a universal influenza vaccine, *e.g.*, how to boost the concentrations of the slower growing cross-reactive antibodies directed to the stem. The results suggest that, upon natural reinfections subsequent to vaccination, the protectiveness of such vaccines would erode, possibly requiring regular boosts. Collectively, our results strongly support the importance of bivalent antibody binding in immunodominance, and suggest guidelines for developing a universal influenza vaccine.

## 1 Introduction

Viral respiratory infections remain a high source of morbidity and mortality around the world. Universal vaccines against highly-mutable viruses such as influenza and HIV are therefore highly desirable. However, their development is complicated by the high antigenic drift of the viral genomes, which stems from low replicative fidelity of viral nucleic acid polymerases. In the case of influenza, there also exists the possibility of antigenic ‘shifts’, whereby genome segments from different viral strains are reassorted in a host co-infected with multiple viruses. Such reassortments can result in pandemic strains against which preexisting immunity in the affected population is low. For example, the H1N1 1918 influenza pandemic was likely caused by a reassortment between avian and human influenza strains, the 2009 H1N1 pandemic was probably caused by a triply reassorted human, avian, and swine strain (1), and the SARS-CoV2 pandemic is believed to have originated from a bat virus adapted to infect humans and other hosts (2).

**Figure 1:**
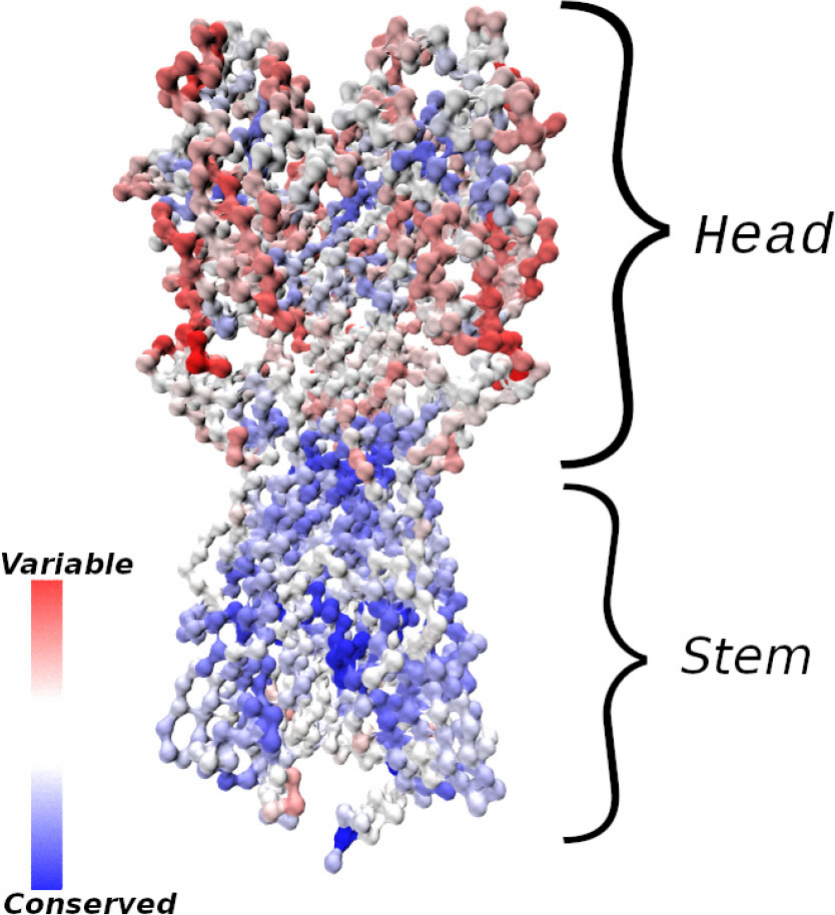
Influenza hemagglutinin (HA) spike protein colored by residue conservation. Sequences of avian, swine and human influenza type A spike proteins spanning the years 1918–2019 and subtypes 1-18 were downloaded from the NIH influenza research database (3); conservation was computed in MATLAB (4) after clustering the sequences to 97% identity and multiple alignment. The HA structure was taken from PDB entry 3LZG (5), and the image was generated using Visual Molecular Dynamics (6). Only the Cα atoms are shown.

Despite the high mutation potential of many viruses, epitopes that are relatively well conserved between different strains, and antibodies that bind to them, have been discovered. In the case of the influenza hemagglutinin (HA) protein (see **Figure 1**), the majority of conserved epitopes are located on the stem or base of the HA (7, 8); less frequently, they are found in the HA head, *e.g.*, at the interface between the heads of HA monomers (9, 10) in a homotrimer, or in the vicinity of the sialic acid receptor (11, 12). There are different plausible reasons for the increased sequence conservation of these regions, such as (i) maintenance of function (*e.g.* the sialic acid receptor is required for host cell entry, and properly folded and probably somewhat rigid HA helices are needed for fusion with the host cell) and (ii) a lack of evolutionary pressure to drive escape mutants (11); the stem epitopes and the occluded epitope (9) are less accessible to antibodies, and thus experience less selective pressure than the exposed HA head. Because antibodies (Abs) that bind conserved epitopes could confer protection against many different viral strains, the elicitation of such cross-reactive, or broadly-neutralizing Abs (bnAbs) has been a goal of vaccine development (7). BnAbs have been isolated from animal and human subjects in response to natural infection or vaccination (8), and some have been engineered (13) to have very high breadth, providing simultaneous protection against phylogenetically distant group I and II HAs (13–15). We note, however, that strong sequence or structural conservation of epitopes shared by different antigens is not an absolute requirement for targeting by bnAbs, as many polyreactive and self-reactive bnAbs have been discovered that are able to bind divergent ligands through structural flexibility (16, 17).

Unfortunately, bnAbs tend to be rare and are not well boosted in infections (15, 18–20). Further, most of them have lower neutralization potency, compared to strain-specific Abs. However, bnAbs have different mechanisms of protection, *e.g.*, labeling infected cells for destruction by Ab-directed cytotoxicity (ADCC), or inhibiting membrane fusion after phagocytosis by the host cell (7), rather than blockage of viral binding to cells, so that direct comparisons between different Abs are not always informative. Many bnAbs have been shown to provide heterosubtypic protection in passive immunization studies (8), indicating that their elicitation by a vaccine could provide broad immunity. In the case of influenza, titers of stem-directed Abs have been shown to increase with age, correlating inversely with incidence of symptomatic infection (21).

The antigenic epitopes targeted by rare bnAbs are labeled immunosubdominant, because they usually are not the primary targets of immune responses. Since durable vaccine responses against highly-mutable pathogens will need to overcome immunodominance, *i.e.* to focus on conserved subdominant epitopes, various possible causes for subdominance have been considered. They include (i) epitope autoreactivity (12), (ii) low frequency of germline precursor antibodies able to bind the epitopes in question (22), which is related to the idea of “holes” in the human antibody repertoire, exploited by HIV to protect its conserved regions (23), (iii) epitope shielding by glycans (10), (iv) preexisting immune memory (20, 22), poor steric accessibility of epitopes (24), (v) inability to recruit sufficient T-cell help in germinal centers (GCs) due to a lack of compatible MHCII (major histocompatibility complex of type 2) epitopes (19), and (vi) entropic dominance of distracting epitopes (24–26). Although multiple effects are likely to contribute, it is of interest to identify the most important ones. In the context of influenza HA, especially in regard to the stem and the occluded interfacial epitope of Watanabe et al. (9), low steric accessibility appears to be a particularly important factor.

Andrews et al. (22) found that some stem-directed Abs bound virus particles with an affinity that was an order of magnitude lower than that for recombinant HA, which was interpreted in terms of reduced accessibility of stem epitopes of whole virions. Harris et al. (27) used cryo-electron tomography (cryo-ET) to estimate the average spacing between HA spikes on influenza virions to be 14nm, which appears to be compatible with bivalent binding of antibodies to the HA stem, based on the docking of an unrelated mouse IgG antibody (27). However, although the study demonstrated that stem epitopes are accessible to antibodies, because of rotational averaging, only one Fab of a stem antibody could be placed based on the cryo-ET data; the authors suggested that steric constraints may lower binding stoichiometry. Amitai et al. (24) used molecular simulations to map the accessibility of HA epitopes to antibody binding on model virus-like particles (VLPs), and predicted that the rate of bivalent (avid) antibody binding to stem epitopes is much lower than that for head epitopes. The importance of bivalent binding in antibody maturation was demonstrated elegantly by Kanekiyo et al. (28), who co-displayed HA receptor binding domains (RBDs) from different influenza strains on nanoparticles (NPs). The heterotypic NPs (RBDs from multiple strains on an NP) elicited Abs with higher breadth than a mixture of homotypic NPs (RBD from a single strain on an NP). Because the vaccines differed only in the geometric arrangement of the antigens, and not in composition or proportion, the results imply that mosaic display confers an advantage to cross-reactive Abs via bivalent binding (28). Similar results were obtained recently for the SARS-CoV2 RBD (29). The importance of multivalent antigen binding in affinity maturation was also shown in the discovery of vaccine-induced Fab-dimerized antibodies directed to HIV glycans (30).

Motivated by these studies, we employed a coarse-grained computer model of affinity maturation (AM) in GCs to investigate how avidity differences in the binding of B-cell receptors (BCRs) to their cognate antigens (AGs) could influence the patterns of immunodominance observed experimentally. Despite the coarse-graining, the model allows comparisons between multiple competing epitope/paratope pairs, in terms of B-cell and Ab production and affinity for antigen. We first show that the model is in qualitative agreement with experimental observations of the basic properties of GC reactions, and of the subdominance of influenza stem epitopes, whose predominant mode of binding to BCR/Ab is assumed to be monovalent. We then use the model to simulate multiple exposures to an antigen and interpret the results to propose strategies for overcoming immunodominance. We suggest that bnAbs that target subdominant epitopes are most likely to be elicited by increasing effective epitope concentration *via* design of custom immunogens or cocktail composition. The present model also predicts the resulting immune memory to be short-lived, which suggests that regular boosts may be required for vaccines composed of immunosubdominant epitopes.

Because the model and its computer implementation involve many technical aspects, we include the detailed *Methods* section at the end of the paper, after the *Results* and *Discussion*, which are presented next.

## 2 Results

**Figure 2:**
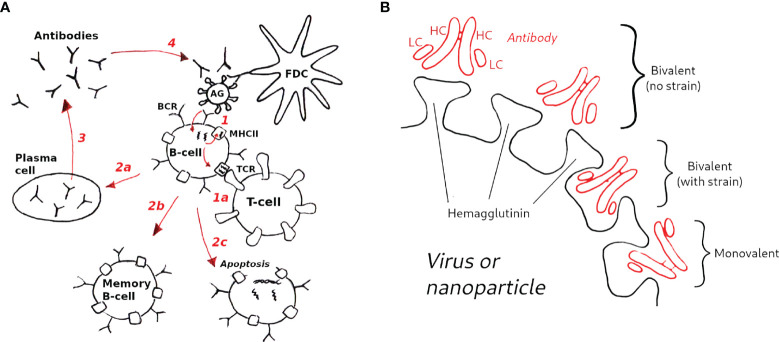
Schematic of the GC model used in this study. **(A)** Model overview (1): B-cells are activated upon binding to antigen presented on follicular dendritic cells (FDCs, not explicitly modeled); (1a) in the optional T-cell help model (see text) B-cells are activated when the major histocompatibility complex receptor (MHCII) binds to the T-cell receptor (TCR); B-cell activation rescues B-cells from apoptosis, allowing them to mutate and proliferate (2); depending on the activation signal, B-cells can differentiate to plasma cells (2a), to memory B-cells (2b), or undergo apoptosis (2c) (3); Plasma cells secrete antibodies (Abs), which also compete with B-cell receptors for antigen (4), which is the essential aspect of the Ab feedback model (31) (see text). **(B)** Hypothetical modes of antibody binding to influenza spikes; bivalent binding without strain (top) corresponds to cooperative binding by antibody arms; bivalent binding with strain (middle) corresponds to noncooperative binding; monovalent binding (bottom) is assumed to be the dominant mode of binding of anti-HA stem antibodies (see *Methods* for details).

A visual schematic of the model is given in **Figure 2**, and a brief overview is presented below. Complete details of the model equations and parametrization are given in *Methods*, and the parameter values used are listed in **Table 1**. Our approach is related to the differential equation models of Kepler and Perelson (32) and listed Oprea and Perelson (33). These models achieve a balance between biological detail and computational complexity: (i) they are sufficiently accurate to model the population sizes of B-cell receptors (BCRs) with different affinities, represented by discretized affinity classes; (ii) they contain a relatively small number of parameters, and (iii) they are simple enough to permit a large number of simulations to study the effects of different initial conditions or model parameters (32, 33). More sophisticated approaches exist, which model individual interactions between immune complexes (ICs), Ags, BCRs and T-cells, aiming to capture GC reactions in higher detail (34, 35). However, their use would be computationally prohibitive for this study. For example, modeling the case in which the initial BCR affinity to an antigen was drawn randomly from a distribution involved 8400 germinal center simulations, each 35 days in duration (see Results).

**Table 1:** Model and simulation parameters.

Parameter	Description	Value	Source
$k_{p}^{\max}$	maximum B-cell proliferation rate	2.2	adjusted from 4(32) to fit data (42)
*B_max_*	Maximum allowed B-cell count	5000	fit to data (42)
*k_d_*	B-cell death rate	4.125	adjusted from 4 (32) to fit data (42)
*μ*	BCR mutation rate	0.1	Oprea and Perelson (33)
σ	BCRs per B-cell	10^5^	Casten and Pierce (62)
*P_L_*	Fraction of lethal mutations	0.5	Kepler and Perelson (32)
*C_M_*	MBC differentiation constant	0.3	fit to data (42, 44)
*C_p_*	PC differentiation constant	0.7	fit to data (42, 44)
$k_{d}^{M}$	MBC death rate	0.02	Rundell et al. (64)
$k_{d}^{a}$	Decay of AG presented to BCRs	0.111	Rundell et al. (64)
$k_{d}^{P}$	PC death rate	0.0336	Rundell et al. (64)
$k_{M}^{P}$	PC production rate from MBCs	0.17	fit to data (44)
$k_{p}^{A}$	AB secretion from PCs	35000	based on Refs (31, 65) (see text)
$k_{d}^{A}$	AB death rate	0.069	Zhang et al. (31)
$k_{d}^{a}$	AG removal rate	0.011	Rundell et al. (64)
*ρ*	activation prefactor in *h* = *ρƟ^ε^*	0.94	fit to data (42, 44)
ε	activation exponent in *h* = *ρƟ^ε^*	0.7	fit to data (42, 44)
ξ	Concentration corresponding to one cell†	1e-4	Kepler and Perelson (32)
*κ^min^*	Smallest (nondimensional) affinity‡	7.5^-2^	Kepler and Perelson (32)
*κ^max^*	Largest (nondimensional) affinity‡	7.5^5^	Kepler and Perelson (32)
Λ	Mutation distribution constant*	≃3.5	based on (32)
*N_E_*	Number of affinity classes	20	up from 8 (32) for higher resolution

Time is measured in days.

^†^Used only for computing cell counts a posteriori, i.e. does not impact simulations;

^‡^The affinity bounds apply only to the first binding constant $\kappa_{j}^{i1}$ ; the second binding constant was defined as different multiples of the first to investigate avidity effects (see Sec. 2);

^∗^The ratio of advantageous to deleterious but nonlethal mutations is given by 1/(1 + Λ^2^); It is computed by logarithmic scaling of the value Λ^0^ = 30 of Kepler and Perelson (32) as log Λ = ΔE log Λ^0^/log 7.5, which accounts for the difference that Kepler and Perelson (32) used an 8-point disretization, or 8 affinity classes (corresponding to ΔE = log 7.5), while we use 20 classes.

As in other models of affinity maturation (36, 37), the interaction between BCRs and AGs is represented by a Langmuir isotherm that uses two equilibrium association constants, which represent binding of the first and second antibody arm. Unlike the more detailed models (24, 38), the present model does not have an explicit structural component, and the effect of bivalency is reflected in the assignment of the association constants. Further, we simplify the biology by not distinguishing between centroblasts and centrocytes, nor between light and dark zones (LZ *vs.* DZ). Instead, we consider the overall proliferation and death rates. This choice is motivated by the findings that the differences between centroblasts and centrocytes, and, more generally between the LZ and DZ, are smaller than previously thought (39); *i.e.*, the physical boundary between LZ and DZ is rather diffuse, selection and proliferation can occur in both zones, albeit with different rates, and T-helper cells appear to be present in both zones, albeit in different proportions. Because we are interested in the dependence of the immune response on the number of competing BCRs with different binding valency and affinity, the model is parametrized to predict the following quantities: (i) the number of GC B-cells, (ii) the number of memory B-cells (MBCs), some of which can be recruited into secondary GCs, (iii) the number of plasma cells (PCs) that secrete Abs, (iv) the number of Abs, which compete with BCRs for antigen and implicitly regulate GC size (31), and, optionally, (v) the T-helper cell population (which is discussed in **Supplementary Material**). The species (i)–(iv) are distributed into affinity classes, while the T-cell model uses two equilibrium affinities, those between TCRs and MHCIIs loaded or unloaded with peptides. The T-cell model is related to the T-cell expansion model of Mayer et al. (40). We consider the model to be optional for the simulations performed here, because qualitatively the same results were obtained by assuming that the amount of T-cell help is equal to BCR activation by binding to antigen. However, the reason for the similar results could be the simplicity of our model, as we did not consider multiple distinct T helper cell populations, which were shown to be important for bnAb elicitation in a recent computational study (41). Thus, in the main text, we present simulation results obtained without an explicit T-cell model. However, several validation calculations performed with the model are described in **Supplementary Material**.

**Figure 3:**
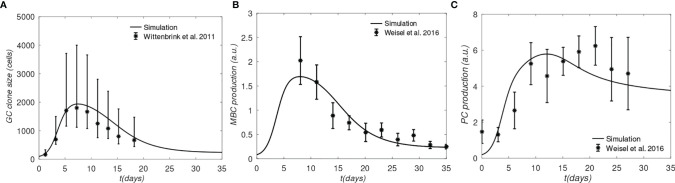
Comparison of simulation and experiments. **(A)** Total B-cells, **(B)** Memory B-cell production rate, **(C)** Plasma cell production rate. Experimental data for panel A was generated from the GC cross-sectional areas plotted in **Figure S1B** of (42), and converted to B cell counts as done in (43); The lower and upper error bars in panel **(A)** corresponds to 30% and 70% quantiles, respectively; experimental data for panels **(B, C)** was taken from **Figure 4** of (43), who obtained raw data from Weisel et al. (44); the error bars in **(B, C)** correspond to approximately one SD.

We first show that the model captures some experimentally determined properties of GCs. In **Figure 3** we compare the model results obtained with a single BCR/Ag pair to the average GC size reported by Wittenbrink et al. (42), and the MBC and PC production rates determined by Weisel et al. (44). The experimental data were also used by Pélissier et al. (43) to parametrize their stochastic GC model, which was used to explore the mechanism of clonal bursts. We note that the Wittenbrink et al. (42) observed very high CG size variability, as reflected in the experimental error bars (**Figure 3**); thus, the agreement between our model and the experimental average should be considered as a qualitative validation, as the model does not capture GC size heterogeneity.

One apparent disagreement is that rate of PC production in our model is accelerated by several days, compared to the data of Weisel et al. (44) (see **Figure 3C**). Although the experimental MBC and PC production in **Figures 3B, C**, respectively, correspond to the same time, matching the PC production rate in the simulation required shifting the experimental measurements by several days (compare **Figure 3B** with **Figure 3C**). However, the discrepancy is not expected to affect our results significantly, because we are interested in comparing the relative MBC output from different lineages at the end of GC reactions, which are simulated consistently, *i.e.* using the same model.

Next, we investigate the effects of BCR binding avidity under different scenarios. First, we compare the growth rates of three noncompeting B-cell lineages, which differ only in the value of the second-arm equilibrium binding constant $K_{eq}^{2}$ (written as *K^i2^* for lineage *i* in Eq. (4) of *Methods*). This idealized scenario corresponds to three GCs evolving independently, which is the noninteracting, *o*=0, case (see *Methods, Sec. 4.1.5*), each completely dominated by a single B-cell lineage.

We compare the behavior of lineages with three regimes of bivalency, corresponding to $K_{eq}^{2}=0,\;K_{eq}^{2}=K_{eq}^{1},\text{ and }K_{eq}^{2}=10\;K_{eq}^{1}$ , which we denote, respectively, as the monovalent, noncooperative, and cooperative binding cases. The justification for the chosen values is discussed in the *Methods Sec. 4.1.2.*

**Figure 4:**
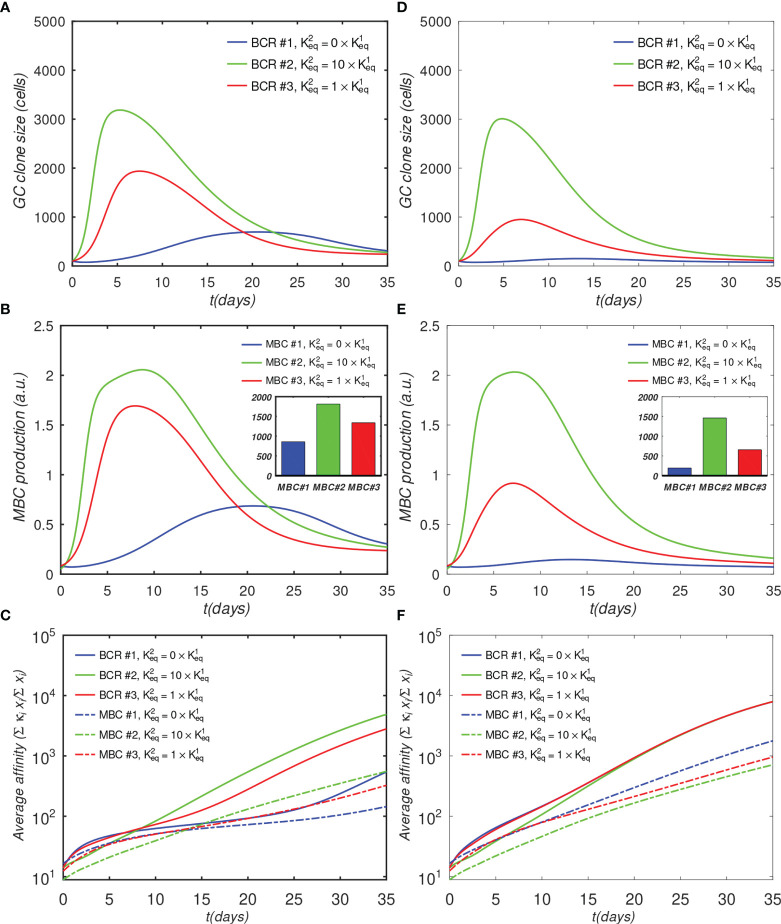
Effect of antibody valency and epitope occlusion on GC properties. Left column **(A–C)** noninteracting B-cell case (*o* = 0); Right column **(D–F)** fully interacting B-cell case (*o* = 1); **(A, D)** Total B cells; **(B, E)** Memory cell production rate; insets: total MBC population at end of simulation; **(C, F)** Average affinity of B-cells and MBCs. For the definition of occlusion o, see *Sec. 4.1.5*.

The results in **Figure 4A** show that the monovalent BCR has a significantly slower rate of growth compared with the bivalent BCRs, due to the reduced activation by Ag assumed by the model. In the monovalent case, the maximum B-cell count is less than a third of that in the bivalent cooperative case, and less than half of that in the noncooperative case; this lower maximum is also reached later; *i.e.*, after 20 days, *vs.* 5 to 7 days. Further, the MBC count at the end of the GC is reduced by half in the monovalent case, compared with the cooperative case (**Figure 4B**), and the final average affinity of the B-cell population is several-fold lower (**Figure 4C**). The delayed peak in the monovalent response is qualitatively consistent with the observations of Tan et al. (19), who noted that the anti-influenza stem Ab response was delayed by a week relative to the overall Ab response, and that the B-cell response was several-fold lower in magnitude.

Although the noninteracting GC case is illustrative, it is idealized, because in affinity maturation there will generally be many different B-cell lineages competing for epitopes on the Ags, or for T-cell help signals. Thus, a more realistic model of a single GC needs to account for competition between different lineages. As described in Sec. 4.1.5 we model this competition using an *occlusion* parameter *o*, with *o* = 0 for the fully noninteracting case, presented above, and *o* = 1 for the fully interacting (competing) case. We note that a similar approach to model clonal competition was used by Yan and Wang (45), who introduced interaction parameters to represent Ag binding interference from Abs produced by earlier generations of B-cells.

We describe the results of the fully interacting case (*o*=1) next; however, we note that a more realistic description of the overall GC reaction would probably involve intermediate values of *o*. For example, if, in some cases, different B-cells are able to bind to different epitopes on the same Ag simultaneously, the occlusion parameter would need to be less than 1, which would correspond to decreased competition between the B-cells. Moreover, when modeling multiple GCs, it may be necessary to include the possibility that the Abs secreted by the plasma cells can diffuse across many GCs, and compete with the ‘local’ BCRs for Ag (31). This scenario would correspond to indirect competition between different B cell lineages in different GCs, which might be modeled with some optimized intermediate (though generally unknown) occlusion value. A possible starting point for estimating such a value could involve competitive binding experiments using antibodies specific for the different epitopes.

The simulation of the fully interacting case shows that the population disadvantage of the monovalent lineage is further increased (**Figures 4** **D–F**). The peak B-cell concentration of the monovalent lineage is more than 10-fold lower than that of the bivalent cooperative lineage (panel D). These results are to be expected, because the more rapidly proliferating lineage occludes the Ag, effectively reducing the amount of epitope available to the monovalent lineage. The average monovalent MBC production is lower by about a factor of eight relative to the bivalent cooperative case (panel E).

It is noteworthy that the average affinities of the three BCRs are indistinguishable after about 20 days after initiation of the GC reaction in the fully competing case (panel F). Further, the BCR affinities in this case at the end of the simulation are higher than those in the *o*=0 case (**Figure 4C** *vs.* **Figure 4F**). This result is understandable in terms of increased competition for survival inside the interacting (*o* = 1) GC. The fact that binding by one BCR occludes access to other epitopes implies that the effective epitope availability is decreased for all BCRs. A decrease in the available binding sites increases the selection pressure on the BCRs, leading to the survival of the higher-affinity lineages. We will return to this point when we investigate the effects of varying epitope concentration explicitly. For completeness, simulation results with intermediate values of occlusion are shown in **Figure S1**.

**Figure 5:**
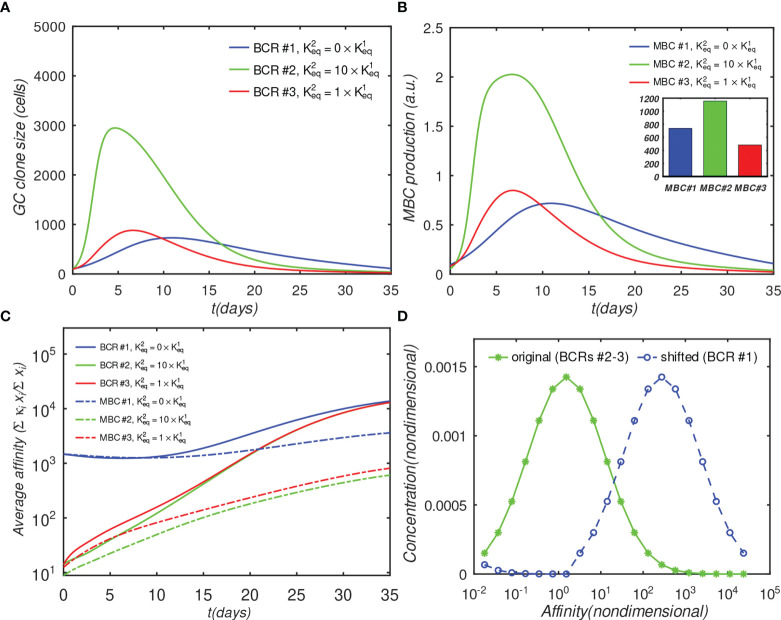
Effect of initial affinity advantage on the growth of monovalent B-cells in the fully interacting B-cell case (*o* = 1). Panels **(A–C)** show the same quantities as **Figures 4A–C**; The affinity distribution corresponding to BCR#1 was shifted toward higher values relative to BCR#2 and BCR#3 panel **(D)**.

As discussed in the introduction, the reason for targeting immunosubdominant epitopes such as the influenza HA stem or the interfacial epitope (9) in vaccinations is their association with the elicitation of bnAbs, which are likely to provide protective immunity against future strains. In the context of the present simulations, such pre-existing protective immunity can be modeled by increasing the initial affinity of the monovalent antibodies, while keeping the other affinities unchanged. This gives the monovalent antibodies a survival advantage. A physiological rationale of setting a higher initial affinity of monovalent Abs could be that a significant proportion of monovalent (*e.g.*, anti-HA stem) MBCs are recruited into secondary GCs, where the higher initial affinity allows the MBC-derived blasts to compete more effectively with naïve bivalent B-cells. A recent study that compared the early plasmablast (PB) response with GC B cells obtained by fine-needle aspiration from vaccinated human subjects found a variable, and sometimes large, clonal overlap (12% - 88%) between B cells in the PB pool and those in the GC, suggesting that substantial recruitment of MBCs into GCs is possible (46). To model this scenario, we shifted the initial affinity distribution of the monovalent Abs by about two orders of magnitude towards higher values (see the distributions in **Figure 5D**), and repeated the simulations for the fully interacting *o*=1 case (the *o* = 0 case is shown in **Figure S2**).

**Figure 5A** shows that the population of the monovalent B-cells increased several-fold as compared with **Figure 4D** (no advantage), such that, at their peak, these B-cells were almost as numerous as the bivalent noncooperative ones. However, even the large affinity advantage was insufficient to overcome the dominance of the cooperatively-binding Abs in terms of the total MBC response, which was still significantly lower in the monovalent case (**Figure 5B**). Nachbagauer et al. (47) found that anti-HA stem immunity can be elicited or boosted upon immunization with chimeric HA constructs with HA heads to which the host is naïve, fused to HA stems against which there is preexisting immunity. In other studies (18, 48), it was reported that boosting with HAs from pandemic, rather than with seasonally-drifted strains, boosted anti-stem immunity more effectively. The authors’ interpretation of the results was that the vaccinations boosted preferentially anti-stem responses derived from MBCs, which were able to outcompete the naïve response to the HA head. Further, Ellebedy et al. (18) also found that immunosubdominance of the stem reemerged after repeat immunization with the same pandemic strain.

**Figure 6:**
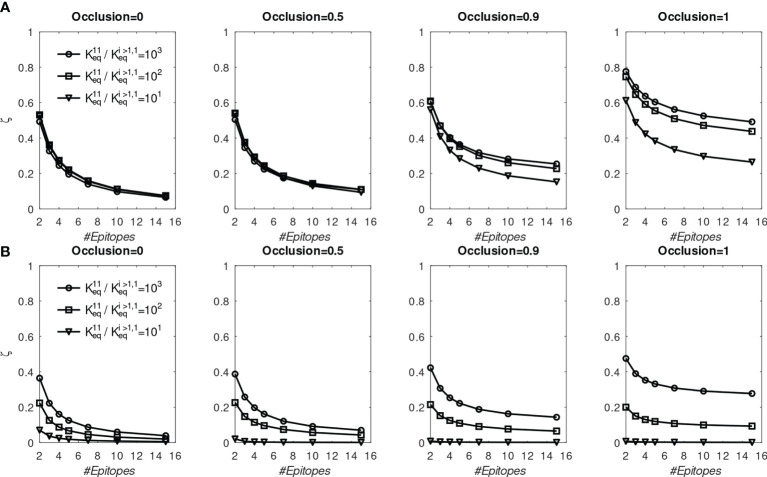
Fraction of MBC#1 (ζ, defined in the text) at the end of six GC simulations for different initial affinity advantage values vs. total number of BCR/Epitope pairs. **(A)** BCR#1 is cooperatively bivalent (K^12^_eq_ =10K^11^_eq_); **(B)** BCR#1 is monovalent (K^12^_eq_ =0).

To test whether the above findings could be explained with the present model, we systematically repeated the preceding simulations for different numbers of distinct BCR/epitope pairs (2 to 15), different occlusion values *o*=[0,0.5,0.9,1], and three different values of affinity advantage provided to BCR#1 (given below); BCR#1 bound monovalently ( $K_{eq}^{12}$ =0) or bivalently $\left(K_{eq}^{12}=10K_{eq}^{11}\right)$ . The remaining BCR#*i* (2 ≤ *i* ≤ 15) were modeled as bivalent with $K_{eq}^{i2}=10K_{eq}^{11}$ . These simulations are discussed below, and their results are shown in **Figure 6**.

The goal of the simulations is to approximate conditions in which one monovalent or bivalent anti-stem BCR (#1) lineage is evolving concurrently with 1-14 bivalent anti-head BCRs. In the first vaccination, all BCRs start from the same affinity distribution peaked at $\kappa_{eq}^{1}≃$ 1.53 (**Figure 5D**). To model the effect of stem conservation after the first vaccination, BCR#1 is given a (multiplicative) affinity advantage over the remaining BCRs of Δ*K_eq_* =10, 100, or 1000. After the first (prime) simulation, each boost is initialized with a combination of 25% MBCs taken at the end of the previous simulation, and 75% naïve B-cells having the same initial distribution as that used for the prime. We assume that the previously-generated anti-head MBCs are poorly matched to the boosting Ag and shift their affinity distribution toward lower values by a factor of 1000, essentially eliminating any advantage of previous maturation. For the presumptive stem-directed BCR#1, we assume that the previously-generated MBCs are better matched to the boosting Ag, and shift their affinity downward only by a factor of 100, 10, or 1 (unchanged), to explore the effect of the mismatch; (thus, the affinity advantage Δ*K_eq_* of BCR#1 corresponds to 1000 divided by 100, 10, or 1). We found that the proportion MBC#1 reaches a plateau by about five immunizations (see **Figure S3**). In **Figure 6** we show the fraction of MBCs#1 after the sixth simulation.

First, we discuss the results of the cooperative bivalent anti-stem case (**Figure 6A**). Here, BCR#1 does not have an avidity disadvantage (since it is cooperatively bivalent with $K_{eq}^{12}=10K_{eq}^{11}$ ), relative to the remaining BCRs, and has an affinity advantage, as described above. In the noncompeting case (*o*=0) the MBC fraction ζ= $MBC_{1}/\Sigma_{i=1}^{N_{B}}MBC_{i}$ is not very sensitive to the affinity advantage, because the Abs are maturing independently and the concentration of each epitope is the same. As the competition between the BCR lineages is increased, the affinity advantage becomes more important. For example, in the high occlusion cases *o* ≥ 0.9, with 9 competing low-affinity BCRs, a 1 to 3 order of magnitude affinity advantage results in ~20% to ~50% of the final MBC population being derived from BCR#1 (ζ∈[0.2,0.5] in the two right panels of **Figure 6A**).

In contrast, the monovalent anti-stem response (**Figure 6B**) produces markedly lower, though still significant MBC#1 proportions. For the lowest initial affinity advantage (×10), the proportion of MBC#1 is vanishingly small for all interacting cases. For the higher advantage values (×100 and ×1000), the proportion of MBC#1 with 9 competing low-affinity BCRs at *o* ≥ 0.9 is in the range ~10% – ~30% (ζ∈[0.1,0.3] in the two right panels of **Figure 4B**).

We interpret these results to suggest that a previous response to a conserved epitope could be boosted to dominate the subsequent response, even in the presence of a significant number of poorly-conserved ‘distracting’ epitopes. This is consistent with the chimeric vaccination results of Nachbagauer et al. (47). However, the extent of boosting is critically dependent on the affinity advantage of the preexisting immunity. In the case of monovalent antibodies, the affinity advantage needs to be high to overcome the proliferation disadvantage caused by monovalency, and the entropic disadvantage caused by distracting epitopes that generally outnumber conserved ones. Ellebedy et al. (18) noted that vaccination with a pandemic strain against which anti-HA head immunity is low, preferentially boosted anti-HA stem antibodies. However, upon reimmunization with the same antigen, the anti-HA head Abs were boosted preferentially. These results can be rationalized in the present model by the differential affinity advantages of the anti-HA stem Abs. Specifically, for the first immunization, the anti-stem immunity has a sufficient affinity advantage to overcome the growth-related and entropic disadvantages. For the second immunization, the affinity advantage is eroded because the anti-HA head immunity has undergone affinity maturation caused by the first immunization, which leads to the restoration of stem epitope subdominance.

Because the affinity advantage of a conserved epitope cannot be predicted accurately if antigenic drift is present, a more realistic approach would be to treat Δ*K_eq_* as a random variable. To investigate this scenario, we performed a round of simulations in which Δ*K_eq_* was sampled from the lognormal distribution; the results are described in **Supplementary Material S1**. The differences between the two cases were similar to those in the simulations with fixed Δ*K_eq_*. For example, in the bivalent case, some MBC#1 cells were always present, while in some simulations of the monovalent case the MBC#1 proportion is near zero (see **Figure S4**).

The above simulations suggest that an affinity advantage alone will not always be sufficient to overcome the disadvantage of slower proliferation and entropic distraction. This result, together with other factors, such as low germline precursor frequency or T-cell help insufficiency (19), could explain the lower prevalence of anti-HA stem immunity.

In natural infections, the epitope concentrations are predetermined by the Ag itself (*e.g.*, the solvent-accessible surface area of the HA head on an influenza virion is about twice that of the stem; see **Figure S5** in **Supplementary Material S2**). However, the vaccination setting allows use of designed antigens with some epitopes masked by glycosylation (10, 49, 50) or even completely removed using protein engineering (51, 52). Additionally, one can administer concurrently multiple Ags, in which some Ag epitopes are sufficiently conserved between the antigens that they can be considered to represent a single epitope with a higher effective concentration (26, 28, 29, 53). In the next set of simulations, we investigate the interplay of epitope concentration and BCR binding valency.

**Figure 7:**
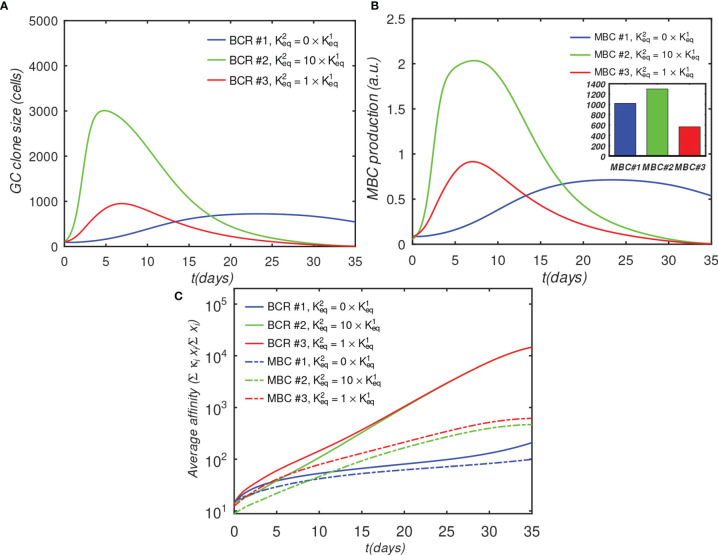
Effect of BCR valency and epitope concentration on GC evolution, with *o* = 1 (fully competitive case). Panels **(A–C)** show the same quantities as **Figures 4A–C**; $\alpha_{1}^{T}=2,\;\alpha_{2}^{T}=1,\;\alpha_{3}^{T}=1$ ; α^T^ is the nondimensional total Ag concentration (see *Sec. 4.1.6*).

**Figure 7** shows the results of a GC simulation involving three competing BCR/Ag pairs, for comparison with the previous 3-BCR simulations (**Figures 4**, **5**); the noninteracting case can be found in **Figure S6**. The concentration of Ag#1, which corresponds to the monovalent BCR#1, was twice that of the other two Ags, while all other parameters were the same as in the original 3-BCR simulation. The peak monovalent B-cell population increases several-fold relative to the uniform concentration case (**Figure 4D**); it is similar in magnitude to that in the affinity-advantaged case of **Figure 5**, but occurs later, at ~ 23 *vs.* ~11 days. This behavior was expected, because, although BCR#1 has an impaired growth rate due to monovalency, it also has more antigen available, allowing it to grow for longer times. Even though the other (bivalent) BCRs are occluding, the higher concentration of Ag#1 overcomes the occlusion disadvantage, albeit with a slow growth rate. The resulting MBC#1 population is much closer to that of the bivalent cooperative case. However, although the greater abundance of Ag#1 amplifies the total BCR#1 response, it also reduces the competition for this Ag, which results in a lower overall affinity of the resulting B-cells (**Figure 7C**).

For completeness, in **Supplementary Material S3** we repeated the above simulation while systematically varying the number of BCR/epitope pairs (2–15), occlusion parameter value (0,0.5,0.9,1), with three values of affinity advantage provided to the BCR#1, and with the BCR#1s bound monovalently or bivalently. The results are summarized in **Figure S7**. In all cases, increasing Ag concentration leads to greater MBC output, with the increase being larger if the corresponding BCR also has a significant affinity advantage. The increased MBC output is associated with decreased affinity, however, and the affinity decrease is larger for monovalent than bivalent BCR#1s. The results therefore suggest that epitope subdominance can be overcome by increasing epitope concentration in vaccinations with cocktails of designed antigens, as proposed by others (26, 28, 29). However, this is achieved at the expense of a reduction in the affinity of the resulting MBCs. For vaccine design, the precise Ag concentrations may need to be optimized to achieve a compromise between MBC population size and affinity for antigen. We caution, however, that the above results should be considered qualitative because our model does not incorporate a saturating Ag concentration, which could be done in future versions, *e.g.*, by explicitly modeling immune complexes or FDCs. Thus, the strong dependence of the B-cell response on the Ag concentration is most likely relevant in a scenario where the total antigen amount is low.

**Figure 8:**
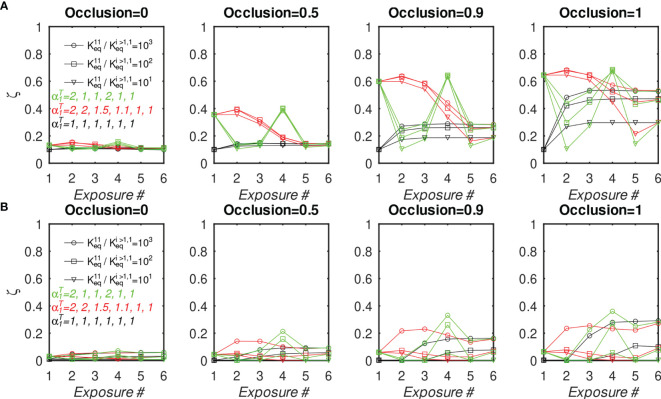
Fraction of MBC#1 vs. number of sequential GC simulations for different initial affinity advantage values and different Ag#1 concentrations, with 10 total BCR/Epitope pairs. **(A, B)** MBC#1 fraction (ζ) at the end of simulations. **(A)** BCR#1 is cooperatively bivalent (K^12^_eq_ =10K^11^_eq_); **(B)** BCR#1 is monovalent (K^12^_eq_ =0). The four sets of panels **(A, B)** show the effect of increasing occlusion from *o*=0 (no competition) to *o*=1 (full competition).

Finally, to investigate whether the outcome of multiple vaccinations can be optimized by manipulating the epitope concentrations corresponding to monovalent BCRs, we simulated six consecutive immunizations under the same initial conditions as described before, except that the concentration of Ag#1 was increased in some, but not all, of the simulations. Specifically, we considered three immunization scenarios, in which the total nondimensional concentration of Ag#1, $\alpha_{1}^{T}$ =[Ag#1/Ag#i≥1], in the six consecutive immunizations was (1, 1, 1, 1, 1, 1) (2, 1, 1, 2, 1, 1), and (2, 2, 1.5, 1.1, 1, 1). These three scenarios were chosen to determine whether increased Ag#1 occurring early in a vaccination regimen would translate into superior responses in later exposures. **Figures 8** and **S8** show the MBC output and affinity, respectively, at the end of each immunization for ten BCR/epitope pairs (other cases are omitted for clarity, but are qualitatively similar). Consistent with the previous results, MBC#1 output after a particular immunization increases if Ag#1 used in that immunization is increased (and vice versa), and the MBC#1 affinity decreases if Ag#1 used in that immunization is increased (and vice versa). However, the differences between the three protocols essentially disappear after the final exposure, hence the present model suggests that there may not be a significant long term immunological effect of simply manipulating antigen concentration in a vaccine.

We also note that, in the idealized case of a uniform antigen concentration profile, the normalized BCR affinities for antigen rise uniformly to a plateau around the 5th exposure (**Figure S8**). However, the results show a sensitivity to the antigen concentration profile, suggesting that the number of exposures needed to elicit high-affinity antibodies depends on the details of the exposure, such as epitope concentration, or whether the exposure is by vaccination or natural infection.

## 3 Discussion

Rapidly mutating and proliferating viruses such as influenza, HIV, and, more recently, SARS-CoV-2, accumulate escape mutations that can render existing host immunity obsolete. However, because such pathogens must maintain infectivity to survive, some mutations are highly improbable, as they would significantly reduce or even eliminate viral fitness. In addition, selection pressure from the immune system is variable along the antigenic sequence (*e.g.* solvent-exposed regions are more susceptible to antibodies than buried ones). The resulting differences in mutation propensities make it possible to partition the viral topology into variable and conserved epitopes. Unfortunately, variable epitopes tend to be immunodominant, *i.e.* they are the main targets of adaptive immunity. Immunodominance, in itself, may be the result of viral adaptation; for example, the large highly-variable head of influenza hemagglutinins is an entropic distraction to the immune system. A major focus of current vaccine research is to elicit a potent and durable immunity to conserved, immunosubdominant, epitopes. Such vaccines would lower HIV infection rates, or eliminate the need for a yearly influenza vaccine.

Here, we employed a coarse-grained model of affinity maturation (AM) parametrized using experimental data on germinal centers (GCs) (42–44) to determine whether differences in B-cell receptor (BCR) binding valency could explain the subdominance of certain epitopes. The main assumption of the model is that B-cell activation increases with the amount of equilibrium-bound BCR to antigen (Ag). More specifically, when both arms (Fabs) of BCR bind the antigen displayed on follicular dendritic cells (FDCs), the probability of internalizing the Ag increases, even if the affinity of each receptor for Ag is weak. This assumption appears to be in accord with the experiments; Arevalo et al. (20) interpreted their vaccination boosting data by suggesting that many weak BCR/Ag interactions are sufficient to activate B-cells. Compelling indirect evidence comes from co-display of different Ags on nanoparticles, which was demonstrated to preferentially elicit broadly-neutralizing Abs. These findings (28, 29) imply that avid bivalent binding confers a proliferation advantage, which is consistent with the present model. However, as B-cell activation by Ags is a complicated process, involving cross-linking of the BCRs, it is not clear to what extent avid binding would increase crosslinking. Future experiments and simulations may be needed to shed more light on the activation process.

The present simulations indicate that monovalent B-cells always grow more slowly than bivalent ones, and are therefore easily dominated by B cells that are able to bind bivalently and cooperatively. When given an initial affinity advantage over bivalent B-cells, as might be expected to occur upon recruitment of monovalent memory cells (MBCs) into secondary GCs, the affinity advantage was often insufficient to overcome the slower growth. The monovalent B cells outcompeted bivalent ones only if the affinity advantage was more than an order of magnitude (see **Figure 6B**). These results are in agreement with influenza vaccination experiments, which show that a boost with a pandemic strain for which the host has little immunity against epitopes in the HA head, produces high anti-HA stem titers; a subsequent boost with the same vaccine elicits anti-HA head Abs (18). We have interpreted these experimental data by assuming that anti-HA stem Abs bind monovalently with a high affinity advantage in the first vaccination, but not the second. In the second vaccination, the bivalent anti-HA head Abs are able to overcome the advantage *via* maturation induced by the first shot.

Rather than relying solely on an affinity advantage, a more robust method to boost monovalent B-cells is to increase the concentration of their cognate epitope(s). The simulations indicate that this approach results in the highest number of monovalent MBCs (see **Figures 7**, **8** in *Sec. 2*). The finding is not surprising, since the presence of foreign antigen is what initiates and sustains GC reactions in the first place. However, because selection among B-cells is driven by competition for Ag, an increase in available Ag will allow lower-affinity BCRs to survive. Thus, although the corresponding monovalent Abs become more numerous with increased epitope concentration, they evolve lower average affinity. When we simulated a subsequent GC reaction initialized with MBCs from such a memory pool, but without the concentration advantage (designated by $\alpha_{1}^{T}=1$ in the results), as would occur in a natural infection, the monovalent B-cell population rapidly decreased, such that after two such consecutive GC reactions, there was no difference when compared to vaccinations in which the concentration of the Ag cognate to the monovalent Ab was never increased (**Figure 8**).

These results suggest that increasing Ag concentration might only provide a temporary advantage to the cognate BCRs, as Ag levels are easy to manipulate in vaccination, but not in infection. Nevertheless, the strategy could prove useful to expand the number of initial low-affinity B-cell lineages targeting rare epitopes against which high-quality B-cell precursors are rare, such as group I and II influenza stem epitopes, as also suggested in a recent computational study of COVID vaccine efficacy (54). If subsequent exposures via natural infection restore immunosubdominance (18), regular vaccine boosting with higher concentrations of subdominant epitopes could be required.

We note that multi-antigen vaccination cocktails have been designed, in which the epitopes that are conserved between the antigens are at effectively higher concentration than variable epitopes (26, 28, 29). In particular, mosaic nanoparticles appear to elicit a broader antibody response in animal experiments, compared to cocktail immunizations (28, 29). However, it remains to be shown whether such vaccines will lead to improved protection against highly mutable pathogens in the clinic. Simulations performed here suggest that such cocktails have promise to elicit Abs to conserved epitopes *via* a concentration advantage. Future experiments are needed to address whether the resulting immunity would persist after multiple rounds of natural infections.

The model used here relies on simple assumptions to show that for different epitopes with similar accessibilities, which can be interpreted as similar effective concentrations, immunosubdominance can be explained by differences in the antibody binding valency. This scenario appears applicable to the case of natural immunity against influenza hemagglutinins, as Harris et al. (27) have shown that most of the trimeric HA spikes are able to bind an anti-stem antibody. The arrangement of the spikes makes it likely that bivalent binding would be disfavored by energetic strain (24, 27). A related scenario applies in the case of HIV, in which low spike density makes bivalent binding unlikely, but antibodies engineered with long linkers that could bind the same trimeric spike bivalently exhibited >100-fold greater potency (55). However, binding valency alone cannot explain immunodominance that arises in vaccination using soluble HA ectodomains because head and stem epitopes would be expected to have similar antibody accessibilities. Therefore, other factors, such as antigen plasticity, low natural germline precursor frequency, repertoire filtering due to self-reactivity, or reduced T-cell help (41), must also contribute. For example, Keating et al. (56) employed several methods of partially inhibiting GC formation in mice, and showed that the proportion of bnAbs in GC-inhibited mice was not increased relative to wild-type mice or untreated mice. These findings were used to argue that the predominant reason for low bnAb prevalence was not competition between antibody lineages within GCs, but rather other factors, such as removal of bnAb precursors due to immune tolerance mechanisms (56). Such factors could also explain why antibodies produced in natural infections such as SARS-Cov-2 tend to target relatively few antigenic epitopes, despite high overall antigen accessibility (57).

Some of the aforementioned factors could be incorporated into the model in an approximate way in future studies. The effects of adjuvants on B-cell activation can be modeled by parametrizing the B-cell activation function *h* (see *Methods*) to include adjuvant concentration, or by incorporating the latter into a T helper cell model. Similar ideas could be used to include the effects of soluble signaling species, such as interleukins or Calcium ions. Further, more sophisticated approaches that explicitly model BCR evolution in sequence space and/or compute BCR/Ag binding affinity using structural models have been developed. For example, Robert et al. (58) approximated B-cell and antigen interactions by discretizing the epitope and paratope on a lattice, and using an empirical inter-residue potential (58). The authors were able to capture key properties of multi-antigen vaccinations, such as increased cross-reactivity in cocktail immunizations. However, BCR/Ag models at all-atom resolution (59, 60), which may be parametrized to account for antigen stability and rigidity, may ultimately be required to design actual vaccine antigens and their cocktails.

For the practical purpose of universal vaccine design, we can summarize the interpretation of our simulation results as follows. HA stem epitopes presented on influenza virions are immuno-subdominant due to an inability to recruit bivalently-binding BCRs, combined with other causes of subdominance. Even if vaccination with soluble antigen ectodomains elicited an anti-stem response, it would not be boosted in secondary GCs formed upon subsequent natural reinfection, because the corresponding B-cells would be unable to bind antigen bivalently. It remains to be shown whether this disadvantage could be overcome by devising vaccines that present stem epitopes for bivalent binding, *e.g.* by using engineered immunogens attached to nanoparticles (52), possibly in a mosaic arrangement (29), or by immunizing with cocktails with very similar stems but diverse heads (26).

While immunization with diverse coronavirus receptor binding domains presented as mosaic nanoparticles elicited a broad antibody response, including to strains not present in the vaccine (29), when this strategy was applied to a diverse panel of influenza HA spikes, the resulting breadth was no greater than that observed with immunizations using homotypic nanoparticle cocktails (61). The interpretation was that the epitopes in the mosaic panel were too dissimilar to allow significant bivalent binding, which suggests that careful tuning of antigen sequence similarity may be needed to elicit broad responses *via* a concentration advantage.

## 4 Methods

### 4.1 Model of Germinal Center Affinity Maturation

The model of affinity maturation (AM) used here is based on the work of Kepler and Perelson (32), who used systems of coupled differential equations to calculate the concentrations of B-cells of different discrete affinities for an antigen (Ag). We have generalized the model to simulate the maturation of multiple B-cell lineages, each binding to its cognate antigen mono- or bivalently, and producing memory B-cells and plasma cells, which secrete antibodies of the same affinity for the cognate Ag. The model does not have any geometric or topological component to represent binding, and, where there is no ambiguity, we sometimes use the terms antigen and epitope interchangeably. We will also sometimes use the abbreviation BCR (B-cell receptor) to refer to B-cells, with the implicit scaling assumption that each B-cell has ~10^5^ BCRs on its surface (62, 63).

We assume that the simulated germinal center(s) (GCs) have been seeded by *N_B_* B-cell lineages *B^i^*, and that each lineage can bind only to its cognate antigen *a_i_*. Though the model is based on the system of differential equations of Kepler and Perelson (32), it has several important additions, notably memory cell, plasma cell, and antibody production. For clarity, we first present a minimal version of the model, which is close to the original B-cell model (32) and describe the modifications in subsequent subsections.

#### 4.1.1 Basic Model

For each B-cell lineage, we explicitly model its binding affinity distribution. Specifically, we assume that (1) the equilibrium binding affinity $K_{eq}^{i}$ of any B-cell *B^i^* derived from the lineage *i* for its cognate antigen *a_i_* is in the range $K_{eq}^{\min}\leq K_{eq}^{i}\leq K_{eq}^{\max}$ and (2) that the affinities can be represented by a discrete set of values, as follows. We assign to each *B^i^* a binding energy index *j ≥ 1* such that


(1) $$\log\;K_{eq}^{\min}\leq (j-1)\Delta E+\log\;K_{eq}^{\min}\leq \log\;K_{eq}^{i}<j\Delta E+\log\;K_{eq}^{\min}\leq \log\;K_{eq}^{\max}$$

Equation  (1) corresponds to a uniform discretization of binding affinities in logarithmic space with energy grid spacing Δ*E*, or exponential discretization in affinity space.

As done by Kepler and Perelson (32), we will refer to the energy bins *j* as *affinity classes*. Their number, *N_E_*, is related to Δ*E* by $\log\;K_{eq}^{\max}-\log\;K_{eq}^{\min}=(N_{E}-1)\Delta E$ . We take *N*_*E*_=20 (see **Table 1**), which implicitly determines Δ*E* once $K_{eq}^{\min}$ and $K_{eq}^{\max}$ are chosen. In the simulations, we only allow values of *K_eq_* that correspond to the bin edges, *i.e.*, $K_{j}^{i}=K_{eq}^{\min}e^{(j-1)\Delta E}$ , *1 ≤ j ≤ N*_*E*_, where, for brevity, we replaced the subscript *eq* by the affinity class index *j*.

To simulate affinity maturation, we compute the time evolution of B-cell populations in each affinity class *j* using


(2) $$\frac{d[B_{j}^{i}]}{dt}=\{-k_{d}(1-h_{j}^{i})-k_{p}\}[B_{j}^{i}]+2k_{p}\sum_{k=1}^{N_{E}}m_{kj}[B_{k}^{i}],\text{ for }1\leq i\leq N_{B},$$

where *k_p_* and *k_d_* are proliferation and death constants, respectively, *m_kj_* is the probability for a BCR *B^i^* in affinity class *k* to transition to affinity class *j via* somatic mutations that take place during AM, $h_{j}^{i}$ is B-cell activation function (discussed below), and *N_B_* is the number of different B-cell lineages.

The class transition probabilities *m_jk_* are assumed to be independent of the lineage *i*, and are defined as


(3) $$m_{jk}=\frac{{\left[\mu (1-p_{L})\right]}^{\left|k-j\right|}}{|k-j|!}\frac{\exp(-\mu )}{1+\Lambda^{2(k-j)}},\;j\neq km_{jj}=1-\sum_{k\neq j}^{N_{E}}m_{jk},$$

where Λ determines the ratio of advantageous to non-lethal deleterious mutations, *p_L_* is the probability of lethal mutations, and *μ* is the probability of an expressed (*i.e.* nonsilent) mutation per generation (32). Kepler and Perelson (32) used an oscillating function for *μ*(*t*) to mimic the effect of interconversion of centroblasts and centrocytes on the mutation rate, which was optimized to maximize a ‘total’ affinity *A(t)* of mature B-cells of a single lineage *i*=1 $\left(A(t)=\Sigma_{j}^{N_{E}}[B_{j}^{1}]K_{j}^{1}\right)$ . We use a constant average mutation rate *μ* =0.1, which is appropriate for comparing growth rates of different BCR lineages within the framework of this coarse-grained model; otherwise, one would need to specify the phases of oscillation for each lineage, which are unknown, and might furthermore be stochastic. The constant value *μ*=0.1 was also used by Oprea and Perelson (33).

The activation function $h_{j}^{i}$ is derived from the proportion of the B cells, $B_{j}^{i}$ , that receive a survival signal *via* binding to antigen and/or helper T-cells (Tfh). In the simpler model, which involves only activation by antigen (Ag), $h_{j}^{i}$ is computed from the equilibrium fraction of receptors $B_{j}^{i}$ bound to the cognate Ag *a_i_*. Because each BCR has two binding arms (Fabs), we assume the binding reactions


(4) $$B_{j}^{i0}+2a_{i}\overset{K_{j}^{i1}}{⇌}B_{j}^{i}(a_{i})+a_{i}\overset{K_{j}^{i2}}{⇌}B_{j}^{i}{\left(a_{i}\right)}_{2},$$

corresponding to sequential binding of the first and second Ags to free B-cells $\left(B_{j}^{i0}\right)$ . From the conservation of total B cells $B_{j}^{i}$ ,


(5) $$[B_{j}^{i0}]+[B_{j}^{i}(a_{i})]+[B_{j}^{i}{\left(a_{i}\right)}_{2}]=[B_{j}^{i}],$$

we have


(6) $$[B_{j}^{i}(a_{i})]=\frac{K_{j}^{i1}[B_{j}^{i}][a_{i}]}{1+K_{j}^{i1}[a_{i}](1+K_{j}^{i2}[a_{i}])}$$

and


(7) $$[B_{j}^{i}{\left(a_{i}\right)}_{2}]=\frac{K_{j}^{i1}K_{j}^{i2}{\left[a_{i}\right]}^{2}}{1+K_{j}^{i1}[a_{i}](1+K_{j}^{i2}[a_{i}])},$$

which allows us to compute the fraction of bound BCR arms (two per BCR), provided that the concentration of free Ag, [*a_i_*], is known, *i.e.*


(8) $$\theta_{j}^{i}=\frac{\left[B_{j}^{i}(a_{i})]+2[B_{j}^{i}{\left(a_{i}\right)}_{2}\right]}{2[B_{j}^{i}]}.$$

In the above, $K_{j}^{i1}$ and $K_{j}^{i2}$ are equilibrium binding constants, corresponding to binding by a first BCR arm and the second, respectively. $K_{j}^{i1}$ is equal to the affinity of the Fab, *i.e.*, $K_{j}^{i1}=K_{j}^{i}$ , and $K_{j}^{i2}$ is taken to be proportional to it, $K_{j}^{i2}=C_{i}K_{j}^{i}$ , where the constant *C_i_* is affinity-independent, and reflects geometry-related factors that influence the binding of the second arm, such as excluded volume (entropy), and deformation strain (energy) required to position the second arm for binding. Monovalent binding corresponds to C_i_ = 0. Other values of *C_i_* used in the simulations were 1 and 10, which are discussed in the next subsection.

To evaluate Eqs. (6) and (7), [*a_i_*] is needed. Writing conservation of total antigen $\left[a_{i}^{T}\right]$ , which is prescribed at the beginning of the GC reaction, and possibly evolves during the reaction, we have


(9) $$[a_{i}]+2\sum_{j=1}^{N_{E}}[B_{j}^{i}]\theta_{j}^{i}=[a_{i}^{T}].$$

We solve Eq. (9) for [*a_i_*] iteratively using the Newton-Raphson method (66).

With $\theta_{j}^{i}$ determined, we can compute the activation $h_{j}^{i}$ . Kepler and Perelson et al. (32) modeled a single lineage and used $h_{j}^{1}=2\theta_{j}^{1}$ with $K_{j}^{12}=0$ , *i.e.*, they treated all BCRs as monovalent. In the present model, we modified the functional form of *h* to match the observations of GC size evolution of Wittenbrink et al. (42), while keeping most of the parameters from the KP93 model (32). Specifically, we defined the activation function as


(10) $$h_{j}^{i}=\rho {\left(\theta_{j}^{i}\right)}^{\varepsilon },$$

and performed least squares optimization to improve agreement with the average GC data (42), to obtain *ρ*=0.94 and ε=0.7 (see **Supplementary Material S4** and **Figure S9**). Because $\theta_{j}^{i}<1$ , the prefactor reduces the activation upper bound to *ρ*; the exponent ε < 1 increases activation for smaller binding fraction values, reducing the competitive advantage of higher affinity BCRs.

Two comments on Eq. (2) are necessary. First, the proliferation is split into two terms (32) to expose the fact that mutations in a cell of lineage $B_{j}^{i}$ during division will lead to the loss of the parent cell and a gain of two daughter cells. Second, the proliferation is not *activated* (*i.e.* not proportional to $h_{j}^{i}$ ). Whether activation by Ag and T-cells mainly rescues B-cells from apoptosis (death rate proportional to [1-*h*]) (36, 67), or actually increases the rates of proliferation (growth rate proportional to *h*) has been a matter of some debate, with more recent evidence in favor of activated proliferation (68). In this study, however, parametrizing the model with activated proliferation would not change the main conclusions; the main difference in the activated proliferation model parameters was that the rate constants $k_{p}^{\max}$ and *k_d_* had to be increased and reduced, respectively, to fit experimental data (see **Supplementary Material S5**). The fact that in the *nonactivated* proliferation model the B-cell death rate constant is higher than the proliferation rate (**Table 1**) reflects the importance of rescue from apoptosis to B-cell survival for this model. Comparison of the activated proliferation model results to the experiments (42, 44) is given in **Figures S10, S11**. A study of the two types of proliferation models was performed by Amitai et al. (69), whose main finding was that activated proliferation reduces clonal diversity.

#### 4.1.2 Avidity of Simulated BCRs

To examine the effect of BCR avidity on the evolution of B cells within the GC, we compare the behavior of lineages with three regimes of bivalency, corresponding to $K_{eq}^{2}=0,K_{eq}^{2}=K_{eq}^{1},\text{ and }K_{eq}^{2}=10K_{eq}^{1}$ , which we denote, respectively, as the monovalent, noncooperative, and cooperative binding cases. We can rationalize the chosen values as follows. The binding constant can be expressed in terms of the free energy difference (Δ*F*) between reactants and products, which is approximately decomposable into rotational, translational (equivalently, concentration) and configurational components (70); Because the two binding arms (Fabs) are identical, we assume that they populate identical conformational ensembles, and therefore their Δ*F* of binding can only differ in the rotational and translational entropies, and, possibly, in the energetic strain needed to move the second Fab into its binding position. The translational entropy penalty of binding of the second Fab (Fab2) will generally be much smaller than that of the first (Fab1), because the volume accessible to unbound Fab2 is restricted by the binding of Fab1 to its epitope, whereas the volume accessible to an unbound BCR is of the order of the GC volume. More specifically, we can estimate the volume available to Fab2 when Fab1 is bound to be the volume occupied by an antibody, which is of the order (10*nm*)^3^ = 10^-24^ *m* ^3^. The effective AG concentration is inversely proportional to this volume (*i.e.* we assume that one antigenic site is available to Fab2, restrained by Fab1). In contrast, when Fab1 is unbound, we take the AG concentration to be inversely proportional to the volume of the GC light zone, approximated as 50% of the GC volume (= 0.5 × [80*μm*]^3^ = 2.56 × 10^-13^ *m* ^3^) and proportional to the number of individual Ags presented on FDCs. Because antigen is generally abundant in GCs (71), we take the number of Ags available to bind BCRs as 1000 times the typical B-cell count in a maturing GC, which is around 2000 from **Figure 3**. The ratio of antigen concentrations for bound *vs.* unbound Fab1 is then 10^24^ × 2.56 × 10^–13^/(1000 × 2000) ≃ 10^5^. The difference in the rotational entropy penalty due to binding is expected to be much smaller, because antibodies appear to be sufficiently flexible to permit considerable independent rotation of the individual Fabs (72). In particular, we expect the difference to be less than an order of magnitude, and neglect this contribution. If energetic strain (*i.e.* Δ*E*) is needed to accommodate binding of Fab2, it will reduce the binding affinity by the factor exp (Δ*E*/[*k_B_T*]). In the absence of experimental data, we assume a strain energy in the range 1-5 kcal/mol, which corresponds to the reduction of $K_{eq}^{2}$ by a factor in the range exp (1/[*k_B_T*]) – exp (5/[*kBT*]) ≃ 5–4160, where *k_B_T* ≃ 0.6 at T=300*K*. The above crude estimates suggest that, even with a substantial antibody strain of 5kcal/mol, a bivalency binding advantage of ×25 would be present. For simplicity, and to include a margin of safety in our results, we assume a slightly lower binding advantage factor of 10, *i.e.* $K_{eq}^{2}$ = 10 $K_{eq}^{1}$ . We also include the monovalent case, $K_{eq}^{2}=0$ , which can also be interpreted as requiring infinite strain energy for Fab2 binding, and an intermediate case $K_{eq}^{2}=K_{eq}^{1}$ , which we label noncooperative.

A possibility that is beyond the scope of this work is to compute strain energy from molecular dynamics simulations of bivalent Ab/Ag binding. However, such simulations are expected to be difficult because of the large sizes of the antibody and antigen molecules involved.

#### 4.1.3 Memory Cell Production

The basic AM model, Eq.  (2), follows the populations of B-cell lineages (*i*) with different affinities (*i*). However, we are also interested in the memory B-cell (MBC) populations produced by different lineages, since these MBCs will be activated in the host upon repeat infections. As in our earlier modeling work (17), we assume that some of the B-cells exit the GC reaction as MBCs or plasma cells (PCs). MBCs are discussed here and PCs, in the next subsection.

Using $M_{j}^{i}$ to denote MBCs of lineage *i* and affinity *j*, the corresponding evolution equation is


(11) $$\frac{d[M_{j}^{i}]}{dt}=C_{M}h_{j}^{i}(1-h_{j}^{i})[B_{j}^{i}]-k_{d}^{M}[M_{j}^{i}],$$

where the first term corresponds to differentiation from GC B-cells, and the second, to apoptosis. The fraction $h_{j}^{i}(1-h_{j}^{i})$ preferentially selects B-cells of intermediate affinity, reflecting the observation that higher-affinity B-cells are more likely to recycle into the dark zone, rather than exit as MBCs (73). The value of *C_M_* is 0.3 (discussed further later) and the death rate constant $k_{d}^{M}$ is 0.02/day (64).

#### 4.1.4 Plasma Cell and Antibody Production

Under the assumption of constant Ag concentration, Eqs. (2) converge to a steady-state solution, in which the GC is composed of highest-affinity B-cells, surviving indefinitely (32). However, it is known that GCs shrink to 5% of their maximum size after about a month (74). While the assumption of constant antigen used in (32) is likely to be unrealistic, Ag consumption in the GC is not the main cause of GC shrinkage. It is known that immune complexes presented by follicular dendritic (and other) cells (39) persist for very long times, which is probably necessary for immune memory maintenance (75).

To account for Ag consumption, we follow Rundell et al. (64) and model it by exponential decay,


(12) $$\frac{d[a_{i}^{T}]}{dt}=-k_{d}^{a}[a_{i}^{T}]$$

with $k_{d}^{a}$ =0.011/day, which corresponds to a half life of about 63 days.

To model GC shrinkage consistently with the experimental observations of GC size (42), we adopt the antibody feedback model of Zhang et al. (31). The essential concept is that some of the maturing B-cells differentiate into PCs, which secrete Abs. These Abs can diffuse throughout the GCs and compete with BCRs for antigen. Once the Abs are sufficiently numerous and of high affinity, the GC shrinks. Although the process of GC shrinkage is probably considerably more complicated, involving regulatory T cells and various signaling molecules, we employ the Ab feedback model here because it requires few additional variables and parameters (see below), and is able to capture GC size evolution over time, as shown here and in (31).

Since antibodies are secreted by PCs, we began with the PC evolution equation


(13) $$\frac{d[P_{j}^{i}]}{dt}=C_{P}h_{j}^{i}(1-h_{j}^{i})[B_{j}^{i}]-k_{d}^{P}[P_{j}^{i}]$$

However, our attempts at fitting this model to the PC production data of Weisel et al. (44) did not yield good agreement. The main reason is that the MBC and PC production rates have different evolution profiles in the experiments (see **Figure 3B** *vs.* **Figure 3C**), with the MBC rate decreasing rapidly, while the PC rate remains essentially constant within the experimental uncertainty. In contrast, our models for the two quantities (Eqs.  (11) and  (13)) are the same, except for the numerical values of the parameters. To improve agreement in the PC rate, we added an additional, semiempirical, source term to Eq.  (13), $k_{M}^{P}[M_{j}^{i}]$ , to mimic low-level differentiation of MBCs activated by immune complexes carrying antigen into late B-blasts, which differentiate into long-lived PCs (64, 76). The resulting PC evolution equation is


(14) $$\frac{d[P_{j}^{i}]}{dt}=C_{P}h_{j}^{i}(1-h_{j}^{i})[B_{j}^{i}]+k_{M}^{P}[M_{j}^{i}]-k_{d}^{P}[P_{j}^{i}],$$

which maintains some PC production even if the B-cell (but not MBC) population vanishes.

In Eq. (14) the value of the death rate constant $k_{d}^{P}$ is 0.0336/day (64), the differentiation constant *C_P_* is 0.7, and the production constant $k_{M}^{P}$ is 0.18/day. *C_P_* and $k_{M}^{P}$ were first set by trial and error and subsequently refined by least squares fitting to reproduce the average GC dynamics. While we could not obtain a biologically-motivated value for $k_{M}^{P}$ , we note that it is about an order of magnitude lower than the B-cell proliferation constant (see **Table 1**), consistent with its role as a lesser source of PCs. However, its value still appears to be unphysically high, especially in comparison to the MBC death rate of 0.02/d [Eq. (11)], possibly reflecting deficiencies or oversimplifications in the differentiation components of the model. For example, the probabilities of a B-cell exiting the GC to differentiate into an MBC *vs.* a PC are kept constant (*C_M_* =0.3 *vs.* *C_P_* =0.7). Recent data suggests that PCs tend to be produced more frequently in later stages of the GC reaction (44), and that a PC is more likely to result than an MBC if the B-cell has higher affinity for antigen, and/or receives more T-cell help (73). However, because quantitative data describing the relative MBC/PC output is scarce and imprecise, we do not implement an affinity dependence in the MBC/PC differentiation choice, and instead use the same preference for B-cells of intermediate affinity in both cases *via* the factor $h_{j}^{i}(1-h_{j}^{i})$ in Eqs.  (11) and (14). More sophisticated affinity-based cell fate decisions are probably needed in these equations. They can be modeled using other functions of $h_{j}^{i}$ , or by introducing other biological species or signaling molecules, as more precise data become available.

Antibodies are secreted by PCs and their removal is modeled with exponential decay


(15) $$\frac{d[A_{j}^{i}]}{dt}=k_{p}^{A}[P_{j}^{i}]-k_{d}^{A}[A_{j}^{i}]$$

The Ab death rate constant $k_{d}^{A}$ is obtained from the half-life of 10d (31), and an approximate secretion $k_{p}^{A}$ rate constant is obtained as follows. We assume that every PC secretes 1.7 ×10^8^ Ab molecules/day (65). However, the Abs are allowed to diffuse freely in and out of GCs, and, assuming that the diffusion is fast enough to establish equilibrium, we scale this rate by the ratio of internal to external volumes corresponding to a single GC. The internal volume is taken as the volume of a sphere of radius 80*μm* (42), and the external volume is taken to be 0.04*mL* (31), which gives $k_{p}^{A}$ =8500/day. Starting from this value, and the PC differentiation parameter *C_P_* =0.5, we used least squares fitting to improve the agreement between the model and the average GC sizes of Wittenbrink et al. (42) (an example of parameter fitting is shown in **Supplementary Material S4**). The optimized values were $k_{p}^{A}$ =35000/day and *C_P_* =0.7, corresponding to higher values of Ab production needed to achieve faster GC shrinkage.

The effect of Ab competition is incorporated by modifying the Ag conservation Eq.  (9) to include binding to Abs,


(16) $$\begin{array}{l} {}[a_{i}]+2\sum_{j=1}^{N_{E}}([A_{j}^{i}/\sigma ]+[B_{j}^{i}])\theta_{j}^{i}=\\ {}[a_{i}]+\sum_{j=1}^{N_{E}}\left[C_{j}^{i}\right]\theta_{j}^{i}=[a_{i}^{T}], \end{array}$$

where we assumed that Abs bind antigen in the same manner as do BCRs, and defined a total receptor concentration $\left[C_{j}^{i}]\equiv 2([A_{j}^{i}/\sigma ]+[B_{j}^{i}]\right)$ ; the scaling factor *σ* = 10^5^ appears because each B-cell $\left(B_{j}^{i}\right)$ is assumed to have 10^5^ BCRs (62), which have the same binding valency as Abs.

At this stage, the model, as written in Eq.  (2), does not have explicit limitations on the maximum GC size. The shape of the B-cell population curve is governed entirely by proliferation, death and competition with Abs. To obtain a close match to the peak in the experimental B-cell count (42), we follow others (33, 64) and introduce a maximum lineage size *B_max_* = 5000 cells. The proliferation rate is modified as follows,


(17) $$k_{p}=k_{p}^{\max}\times (1-\frac{\Sigma_{j}[B_{j}]}{B_{\max}}),$$

where $k_{p}^{\max}$ is the maximum proliferation rate. A similar idea was used by Amitai et al. (24), who increased the cell death rate, as a critical B-cell population was approached.

#### 4.1.5 Clonal Competition *via* Epitope Occlusion

Thus far, we have described a model which has competition only within each clonal lineage, *i.e.*, higher-affinity cells outcompete lower-affinity cells, and are themselves eventually outcompeted by growing numbers of high-affinity Abs derived from them. However, GCs are seeded by multiple lineages, and it is therefore important to consider the effects of interclonal competition. In the context of influenza, it would be of interest to model how anti-HA-head Abs could directly compete with anti-HA-stem Abs.

Toward this end, we generalize the model by introducing a distinction between epitopes and antigens. Specifically, we recognize that a single antigen can present different epitopes. For example, an entire viral spike may be considered an antigen with many different epitopes, each targeted by a different activated B cell. We postulate that the binding of a BCR or Ab of type *k* to its cognate epitope *a_k_* can reduce the accessibility of epitope *a_i_*, so that the effective concentration of *a_i_* available to bind is reduced by a fraction of the bound concentration of *a_k_*. We label this reduction Δ*^k^* [*a_i_*], which is


(18) $$\Delta^{k}[a_{i}]=-O_{ik}\sum_{j=1}^{N_{E}}\left[C_{j}^{k}\right]\theta_{j}^{k},\text{ with }0\leq O_{ik}\leq 1,i\neq j,$$

where we introduced the *occlusion* tensor *O_ik_*, which models the effect of epitope *k* occupancy on epitope *i*. In particular, *O_ik_* =0 corresponds to the absence of interaction, and *O_ik_* =1 implies that binding of *a_k_* completely prevents binding to *a_i_* (full occlusion). Setting *O_ii_ =* 1, we write the modified Ag conservation equations as


(19) $$[a_{i}]+\sum_{j=1}^{N_{E}}\sum_{k=1}^{N_{B}}O_{ik}[C_{j}^{k}]\theta_{j}^{k}=[a_{i}^{T}],$$

in which the concentrations [*a_i_*] are now coupled *via* the occlusion tensor [unlike in Eq. (16), in which they are independent]. The components of *O* can in principle be set independently, provided that care is taken to avoid component values so large that negative concentrations could result. For simplicity, we begin with a constant occlusion independent of the antigen identity, *i.e.*, *O_ik_* = *o*, for 0 ≤ *o* ≤ 1, and *I* ≠ *k*, and reduce its value to prevent negative AG concentrations. Specifically, if $\left[a_{k}^{T}]>[a_{i}^{T}\right]$ we set


(20) $$O_{ik}=o\times \min\left\{1,\frac{\left[a_{i}^{T}\right]}{\left[a_{k}^{T}\right]}\right\}.$$

In the simplified case of two BCR/epitope pairs, Eq.  (20) can be justified as follows. We combine the conservation equations,


(21) $$[a_{1}]+\sum_{j=1}^{N_{E}}\left[C_{j}^{1}\right]\;\theta_{j}^{1}+O_{12}[C_{j}^{2}]\;\theta_{j}^{2}=[a_{1}^{T}],[a_{2}]+\sum_{j=1}^{N_{E}}\left[C_{j}^{2}\right]\;\theta_{j}^{2}+O_{21}[C_{j}^{1}]\;\theta_{j}^{1}=[a_{2}^{T}],$$

to obtain


(22) $$[a_{1}]+(1-O_{12}O_{21})\sum_{j=1}^{N_{E}}[C_{j}^{1}]\theta_{j}^{1}=[a_{1}^{T}]-O_{12}([a_{2}^{T}]-[a_{2}]).$$

Because 1 – *O*_12_ *O*_21_ ≥ 0 and the bound fraction *θ* ^1^ is zero only if [*a*_1_] = 0 (we assume that the binding constants are not both zero), to ensure [*a*_1_] ≥ 0, it is sufficient to require


(23) $$[a_{1}^{T}]-O_{12}([a_{2}^{T}]-[a_{2}])\geq 0,$$

or


(24) $$O_{12}\leq \frac{\left[a_{1}^{T}\right]}{\left[a_{2}^{T}]-[a_{2}\right]}$$

Eq.  (20) satisfies this condition, since we also assume that [*a*_2_] is nonnegative.

We note that the occlusion tensor is similar in spirit to the interaction matrix used by Yan and Wang (45). However, as these authors had a different purpose, specifically, to model synergistic *vs.* antagonistic effects of Abs derived from previous B cell lineages on B cells in the current generation, they allowed negative interference values, which are not physically justifiable in our model, as they would imply creation of Ag.

#### 4.1.6 Integration of Model Equations

In this section we describe the numerical procedures used to compute the time-dependent concentrations of the Ags, cells, and Abs in the GC. First, following Kepler and Perelson (32), we make all concentrations nondimensional using the total concentration of one of the antigens at the beginning of simulation. For single-epitope (validation) simulations, we use the sole epitope. For multi-epitope simulations, we arbitrarily elected to use the second epitope, to be able to vary the concentrations of the first epitope for studying the effects of epitope concentration in maturation. Thus, the nondimensional variables are $\alpha_{i}\equiv [a_{i}]/[a_{2}^{T}],\;x_{j}^{i}\equiv [B_{j}^{i}]/[a_{2}^{T}],\;y_{j}^{i}\equiv [A_{j}^{i}]/[a_{2}^{T}],\;p_{j}^{i}\equiv [P_{j}^{i}]/[a_{2}^{T}],\;w_{j}^{i}\equiv [M_{j}^{i}]/[a_{2}^{T}],$ and $\kappa_{j}^{ik}=K_{j}^{ik}[a_{2}^{T}]$ .

In the new variables, the nondimensional evolution equations are


(25) $$\frac{dx_{j}^{i}}{dt}=\left\{-k_{d}(1-h_{j}^{i})-k_{p}\right\}x_{j}^{i}+2k_{p}\sum_{k=1}^{N_{E}}m_{kj}x_{k}^{i},$$

(26) $$\frac{dw_{j}^{i}}{dt}=C_{M}h_{j}^{i}(1-h_{j}^{i})x_{j}^{i}-k_{d}^{M}w_{j}^{i},$$

(27) $$\frac{dp_{j}^{i}}{dt}=C_{P}h_{j}^{i}(1-h_{j}^{i})x_{j}^{i}+k_{M}^{P}w_{j}^{i}-k_{d}^{P}x_{j}^{i}$$

(28) $$\frac{dy_{j}^{i}}{dt}=k_{p}^{A}p_{j}^{i}-k_{d}^{A}y_{j}^{i}$$

(29) $$\frac{d\alpha_{i}^{T}}{dt}=-k_{d}^{a}\alpha_{i}^{T},\text{ for }1\leq i\leq N_{B},1\leq j\leq N_{\in }$$

and the nondimensional AG conservation equations are


(30) $$\alpha_{i}+2\sum_{j=1}^{N_{E}}\sum_{k=1}^{N_{B}}O_{ik}(x_{j}^{k}+\sigma^{-1}y_{j}^{k})\theta_{j}^{k}=\alpha_{i}^{T},$$

(31) $$\theta_{j}^{i}=\frac{1}{2}\cdot \frac{\kappa_{j}^{i1}x_{j}^{i}(1+2\kappa_{j}^{i2}x_{j}^{i})}{1+\kappa_{j}^{i1}x_{j}^{i}(1+\kappa_{j}^{i2}x_{j}^{i})}$$

(32) $$O_{ik}=o\times \min\left\{1,\frac{\alpha_{i}^{T}}{\alpha_{k}^{T}}\right\}$$

and the expressions for *m_ij_* and *h* are the unchanged.

We follow Kepler and Perelson (32) and take the dimensionless affinities *κ^min^* = 7.5^-2^, *κ^max^* = 7.5^5^, but use *N_E_* =20 affinity bins, compared to their 8, for a finer discretization. This corresponds to Δ*E*≃0.74 in Eq.  (1). Eq.  (25) – Eq.  (29) were integrated in Octave (77) using the explicit Euler method (78), with the time step *dt* = 0.01× day. At each iteration, Eq.  (30) was solved for *α_i_* using the iterative Newton-Raphson (NR) method (66). The initial guess at the current iteration was taken as the corresponding value in the previous iteration, which ensured that convergence required only a few NR iterations. At the beginning of the simulation, the initial guesses were $\alpha_{i}=\alpha_{i}^{T}$ . Simulation duration was 35 days, requiring less than a minute of computer time using a single computing node with an Intel Xeon E5 2.3 GHz Haswell CPU. However, the simulation cost was approximately linearly dependent on the number of BCR/Ag pairs simulated. The simulation parameters and their values are listed in **Table 1**.

#### 4.1.7 Initial Conditions

Initial values correspond to time *t* = 0. For single-epitope simulations $\alpha_{1}^{T}$ = 1. For multi-epitope simulations, $\alpha_{2}^{T}$ = 1, by definition of the normalization and $\alpha_{i}^{T}$ = 1 for *i* > 2. The concentration of the first epitope $\alpha_{1}^{T}$ was varied between 1 and 2, depending on simulation to investigate the effects of AG concentration (see Results). The initial population of each B-cell lineage was 100 cells, because we assumed that a mutation-free expansion of each seeding B-cell has already taken place prior to simulation. The initial distribution of naive B-cell binding constants was log-normal; specifically, we used a Gaussian in the energy space, centered on the 7th class (*κ* ≃ 1.53) with standard deviation σ = 0.6 × Λ. This choice was made to approximate by a smooth function the discrete Dirac mass used by Kepler and Perelson (32). The distribution is shown in **Figure 4D** of the Results. The above distribution appears to us to be more physical than the sharply peaked distribution of Kepler and Perelson (32). However, we note that we could also obtain a satisfactory fit to the experimental data using the latter distribution, albeit with small parameter adjustments that affect the total GC size and time to GC peak.

For simulations whose purpose was to investigate the effect of an initial affinity advantage on the rate of B-cell proliferation (see Sec. 2), the initial distribution was shifted by scaling the abscissa by a prescribed advantage (Δ*K_eq_*), and linearly reinterpolated onto the initial grid in energy space (see Results and **Figure 6D**). All initial distributions (of each lineage) were normalized to 100 B-cells.

## Data Availability Statement

The datasets presented in this study can be found in online repositories. The names of the repository/repositories and accession number(s) can be found in the article/**Supplementary Material**.

## Author Contributions

VO conceived the research. VO performed the simulations. VO and MK analyzed results and wrote the paper. Both authors contributed to the article and approved the submitted version.

## Funding

Financial support for this project was provided by the Bill & Melinda Gates Foundation and Flu Lab under grant opportunity OPP1214161. The findings and conclusions contained within are those of the authors and do not necessarily reflect positions or policies of the Bill & Melinda Gates Foundation.

## Conflict of Interest

The authors declare that the research was conducted in the absence of any commercial or financial relationships that could be construed as a potential conflict of interest.

## Publisher’s Note

All claims expressed in this article are solely those of the authors and do not necessarily represent those of their affiliated organizations, or those of the publisher, the editors and the reviewers. Any product that may be evaluated in this article, or claim that may be made by its manufacturer, is not guaranteed or endorsed by the publisher.

## References

[1] Taubenberger J Kash J Morens D. The 1918 Influenza Pandemic: 100 Years of Questions Answered and Unanswered. Sci Trans Med (2019) 11:1–15. doi: 10.1126/scitranslmed.aau5485 PMC11000447 31341062

[2] Andersen K Rambaut A Lipkin W Holmes E Garry R. The Proximal Origin of SARS-CoV-2. Nat Med (2020) 26:450–2. doi: 10.1038/s41591-020-0820-9 PMC7095063 32284615

[3] Bao Y Bolotov P Dernovoy D Kiryutin B Zaslavsky L Tatusova T. The Influenza Virus Resource at the National Center for Biotechnology Information. J Virol (2008) 82:596–601. doi: 10.1128/JVI.02005-07 17942553 PMC2224563

[4] MATLAB. Version 7.10.0 (R2010a) (Natick, Massachusetts: The MathWorks Inc.). (2010).

[5] Xu R Ekiert D Krause J Hai R Crowe J Wilson I. Structural Basis of Preexisting Immunity to the 2009 H1N1 Pandemic Influenza Virus. Science (2010) 328:357–60. doi: 10.1126/science.1186430 PMC2897825 20339031

[6] Humphrey W Dalke A Schulten K. VMD - Visual Molecular Dynamics. J Mol Graph (1996) 14:33–8. doi: 10.1038/ni.3684 8744570

[7] Nachbagauer R Choi A Hirsh A Margine I Iida S Barrera A. Defining the Antibody Cross- Reactome Directed Against the Influenza Virus Surface Glycoproteins. Nat Immunol (2017) 18:464–73. doi: 10.1038/ni.3684 PMC5360498 28192418

[8] Corti D Cameroni E Guarino B Kallewaard N Zhu Q Lanzavecchia A. Tackling Influenza With Broadly Neutralizing Antibodies. Curr Opin Virol (2017) 24:60–9. doi: 10.1016/j.coviro.2017.03.002 PMC7102826 28527859

[9] Watanabe A McCarthy K Kuraoka M Schmidt A Adachi Y Onodera T. Antibodies to a Conserved Influenza Head Interface Epitope Protect by an IgG Subtype-Dependent Mechanism. Cell (2019) 177:1124–35.e16. doi: 10.1016/j.cell.2019.03.048 31100267 PMC6825805

[10] Bajic G Maron M Adachi Y Onodera T McCarthy K McGee C. Influenza Antigen Engineering Focuses Immune Responses to a Subdominant But Broadly Protective Viral Epitope. Cell Host Microbe (2019) 25:827–35.e6. doi: 10.1016/j.chom.2019.04.003 31104946 PMC6748655

[11] Raymond D Bajic G Ferdman J Suphaphiphat P Settembre E Moody M. Conserved Epitope on Influenza-Virus Hemagglutinin Head Defined by a Vaccine-Induced Antibody. Proc Natl Acad Sci USA (2018) 115:168–73. doi: 10.1073/pnas.1715471115 PMC5776812 29255041

[12] Bajic G van der Poel C Kuraoka M Schmidt A Carroll M Kelsoe G. Autoreactivity Profiles of Influenza Hemagglutinin Broadly Neutralizing Antibodies. Sci Rep (2019) 9:3492. doi: 10.1038/s41598-019-40175-8 30837606 PMC6401307

[13] Kallewaard N Corti D Collins P Neu U McAuliffe J Benjamin E. Structure and Function Analysis of an Antibody Recognizing All Influenza a Subtypes. Cell (2016) 166:596–608. doi: 10.1016/j.cell.2016.05.073 27453466 PMC4967455

[14] Corti D Voss J Gamblin S Codoni G Macagno A Jarrossay D. A Neutralizing Antibody Selected From Plasma Cells That Binds to Group 1 and Group 2 Influenza A Hemagglutinins. Sci (New York NY) (2011) 333:850–6. doi: 10.1126/science.1205669 21798894

[15] Li G Chiu C Wrammert J McCausland M Andrews S Zheng N. Pandemic H1N1 Influenza Vaccine Induces a Recall Response in Humans That Favors Broadly Cross-Reactive Memory B Cells. Proc Natl Acad Sci USA (2012) 109:9047–52. doi: 10.1073/pnas.1118979109 PMC3384143 22615367

[16] Prigent J Jarossay A Planchais C Eden C Dufloo J Kök A. Conformational Plasticity in Broadly Neutralizing Hiv-1 Antibodies Triggers Polyreactivity. Cell Rep (2018) 23:2568–81. doi: 10.1016/j.celrep.2018.04.101 PMC5990490 29847789

[17] Ovchinnikov V Louveau J Barton J Karplus M Chakraborty A. Role of Framework Mutations and Antibody Flexibility in the Evolution of Broadly Neutralizing Antibodies. eLife (2018) 7:1–24. doi: 10.7554/eLife.33038 PMC5828663 29442996

[18] Ellebedy A Krammer F Li G Miller M Chiu C Wrammert J. Induction of Broadly Cross-Reactive Antibody Responses to the Influenza Ha Stem Region Following H5N1 Vaccination in Humans. Proc Natl Acad Sci USA (2014) 111:13133–8. doi: 10.1073/pnas.1414070111 PMC4246941 25157133

[19] Tan H Jegaskanda S Juno J Esterbauer R Wong J Kelly H. Subdominance and Poor Intrinsic Immunogenicity Limit Humoral Immunity Targeting Influenza Ha Stem. J Clin Invest (2019) 129:850–62. doi: 10.1172/JCI123366 PMC6355240 30521496

[20] Arevalo C Le Sage V Bolton M Eilola T Jones J Kormuth K. Original Antigenic Sin Priming of Influenza Virus Hemagglutinin Stalk Antibodies. Proc Natl Acad Sci USA (2020) 117:17221–7. doi: 10.1073/pnas.1920321117 PMC7382271 32631992

[21] Nachbagauer R Choi A Izikson R Cox M Palese P Krammer F. Age Dependence and Isotype Specificity of Influenza Virus Hemagglutinin Stalk-Reactive Antibodies in Humans. mBio (2016) 7:e01996–15. doi: 10.1128/mBio.01996-15 PMC4725014 26787832

[22] Andrews S Huang Y Kaur K Popova L Ho I Pauli N. Immune History Profoundly Affects Broadly Protective B Cell Responses to Influenza. Sci Trans Med (2015) 7:316ra192. doi: 10.1126/scitranslmed.aad0522 PMC4770855 26631631

[23] Xiao X Chen W Feng Y Zhu Z Prabakaran P Wang Y. Germline-Like Predecessors of Broadly Neutralizing Antibodies Lack Measurable Binding to Hiv-1 Envelope Glycoproteins: Implications for Evasion of Immune Responses and Design of Vaccine Immunogens. Biochem Biophys Res Commun (2009) 390:404–9. doi: 10.1016/j.bbrc.2009.09.029 PMC2787893 19748484

[24] Amitai A Sangesland M Barnes R Rohrer D Lonberg N Lingwood D. Defining and Manipulating B Cell Immunodominance Hierarchies to Elicit Broadly Neutralizing Antibody Responses Against Influenza Virus. Cell Syst (2020) 11:573–88.e9. doi: 10.1016/j.cels.2020.09.005 33031741 PMC7746579

[25] Wang S. Optimal Sequential Immunization Can Focus Antibody Responses Against Diversity Loss and Distraction. PloS Comput Biol (2017) 13:1–27. doi: 10.1371/journal.pcbi.1005336 PMC5279722 28135270

[26] Ives S Bürckert JP Pettus C Peñate K Hovde R Bayless N. A General Solution to Broad-Spectrum Vaccine Design for Rapidly Mutating Viruses. submitted (2020) 1:1–22. doi: 10.21203/rs.3.rs-100459/v1

[27] Harris A Meyerson J Matsuoka Y Kuybeda O Moran A Bliss D. Structure and Accessibility of Ha Trimers on Intact 2009 H1N1 Pandemic Influenza Virus to Stem Region-Specific Neutralizing Antibodies. Proc Natl Acad Sci USA (2013) 110:4592–7. doi: 10.1073/pnas.1214913110 PMC3607006 23460696

[28] Kanekiyo M Joyce M Gillespie R Gallagher J Andrews S Yassine H. Mosaic Nanoparticle Display of Diverse Influenza Virus Hemagglutinins Elicits Broad B Cell Responses. Nat Immunol (2019) 20:362–72. doi: 10.1038/s41590-018-0305-x PMC6380945 30742080

[29] Cohen A Gnanapragasam P Lee Y Hoffman P Ou S Kakutani L. Mosaic Nanoparticles Elicit Cross-Reactive Immune Responses to Zoonotic Coronaviruses in Mice. Sci (New York NY) (2021) 371:735–41. doi: 10.1126/science.abf6840 PMC7928838 33436524

[30] Williams W Meyerhoff R Edwards R Li H Manne K Nicely N. Fab-Dimerized Glycan823 Reactive Antibodies Are a Structural Category of Natural Antibodies. Cell (2021) 184:2955–72.e25. doi: 10.1016/j.cell.2021.04.042 34019795 PMC8135257

[31] Zhang Y Meyer-Hermann M George L Figge M Khan M Goodall M. Germinal Center B Cells Govern Their Own Fate *Via* Antibody Feedback. J Exp Med (2013) 210:457–64. doi: 10.1084/jem.20120150 PMC3600904 23420879

[32] Kepler T Perelson A. Somatic Hypermutation in B Cells: An Optimal Control Treatment. J Theor Biol (1993) 164:37–64. doi: 10.1006/jtbi.1993.1139 8264243

[33] Oprea M Perelson AS. Somatic Mutation Leads to Efficient Affinity Maturation When Centrocytes Recycle Back to Centroblasts. J Immunol (1997) 158:5155–62. 9164931

[34] Meyer-Hermann M. Mathematical Model for the Germinal Center Morphology and Affinity Maturation. J Theor Biol (2002) 216:273–300. doi: 10.1006/jtbi.2002.2550 12183119

[35] Meyer-Hermann M Mohr E Pelletier N Zhang Y Victora G Toellner K. A Theory of Germinal Center B Cell Selection, Division, and Exit. Cell Rep (2012) 2:162–74. doi: 10.1016/j.celrep.2012.05.010 22840406

[36] Zhang J Shakhnovich E. Optimality of Mutation and Selection in Germinal Centers. PloS Comput Biol (2010) 6:e1000800. doi: 10.1371/journal.pcbi.1000800 20532164 PMC2880589

[37] Wang S Mata-Fink J Kriegsman B Hanson M Irvine D Eisen H. Manipulating the Selection Forces During Affinity Maturation to Generate Cross-Reactive HIV Antibodies. Cell (2015) 160:785–97. doi: 10.1016/j.cell.2015.01.027 PMC4357364 25662010

[38] Robert P Marschall A Meyer-Hermann M. Induction of Broadly Neutralizing Antibodies in Germinal Centre Simulations. Curr Opin Biotech (2018) 51:137–45. doi: 10.1016/j.copbio.2018.01.006 29414440

[39] Allen C Okada T Cyster J. Germinal-Center Organization and Cellular Dynamics. Immunity (2007) 27:190–202. doi: 10.1016/j.immuni.2007.07.009 17723214 PMC2242846

[40] Mayer A Zhang Y Perelson A Wingreen N. Regulation of T Cell Expansion by Antigen Presentation Dynamics. Proc Natl Acad Sci USA (2019) 116:5914–9. doi: 10.1073/pnas.1812800116 PMC6442601 30850527

[41] Erwin S Childs L Ciupe S. Mathematical Model of Broadly Reactive Plasma Cell Production. Sci Rep (2020) 10:3935. doi: 10.1038/s41598-020-60316-8 32127549 PMC7054388

[42] Wittenbrink N Klein A Weiser A Schuchhardt J Or-Guil M. Is There a Typical Germinal Center? A Large-Scale Immunohistological Study on the Cellular Composition of Germinal Centers During the Hapten-Carrier-Driven Primary Immune Response in Mice. J Immunol (Baltimore Md 1950) (2011) 187:6185–96. doi: 10.4049/jimmunol.1101440 22102720

[43] Pélissier A Akrout Y Jahn K Kuipers J Klein U Beerenwinkel N. Computational Model Reveals a Stochastic Mechanism Behind Germinal Center Clonal Bursts. Cells (2020) 9:1–25. doi: 10.3390/cells9061448 PMC7349200 32532145

[44] Weisel F Zuccarino-Catania G Chikina M Shlomchik M. Temporal Switch in the Germinal Center Determines Differential Output of Memory B and Plasma Cells. Immunity (2016) 44:116–30. doi: 10.1016/j.immuni.2015.12.004 PMC4724390 26795247

[45] Yan L Wang S. Shaping Polyclonal Responses *via* Antigen-Mediated Antibody Interference. iScience (2020) 23:101568. doi: 10.1016/j.isci.2020.101568 33083735 PMC7530306

[46] Turner J Zhou J Han J Schmitz A Rizk A Alsoussi W. Human Germinal Centres Engage Memory and Naive B Cells After Influenza Vaccination. Nature (2020) 586:127–32. doi: 10.1038/s41586-020-2711-0 PMC7566073 32866963

[47] Nachbagauer R Liu W Choi A Wohlbold T Atlas T Rajendran M. A Universal Influenza Virus Vaccine Candidate Confers Protection Against Pandemic H1N1 Infection in Preclinical Ferret Studies. NPJ Vaccines (2017) 2:26. doi: 10.1038/s41541-017-0026-4 29263881 PMC5627297

[48] Krammer F Pica N Hai R Tan G Palese P. Hemagglutinin Stalk-Reactive Antibodies Are Boosted Following Sequential Infection With Seasonal and Pandemic H1N1 Influenza Virus in Mice. J Virol (2012) 86:10302–7. doi: 10.1128/JVI.01336-12 PMC3457330 22787225

[49] Eggink D Goff P Palese P. Guiding the Immune Response Against Influenza Virus Hemagglutinin Toward the Conserved Stalk Domain by Hyperglycosylation of the Globular Head Domain. J Virol (2014) 88:699–704. doi: 10.1128/JVI.02608-13 24155380 PMC3911724

[50] Zhou T Doria-Rose N Cheng C Stewart-Jones G Chuang G Chambers M. Quantification of the Impact of the HIV-1-Glycan Shield on Antibody Elicitation. Cell Rep (2017) 19:719–32. doi: 10.1016/j.celrep.2017.04.013 PMC5538809 28445724

[51] Yassine H Boyington J McTamney P Wei C Kanekiyo M Kong W. Hemagglutinin-Stem Nanoparticles Generate Heterosubtypic Influenza Protection. Nat Med (2015) 21:1065–70. doi: 10.1038/nm.3927 26301691

[52] Corbett K Moin S Yassine H Cagigi A Kanekiyo M Boyoglu-Barnum S. Design of Nanoparti880 Culate Group 2 Influenza Virus Hemagglutinin Stem Antigens That Activate Unmutated Ancestor B Cell Receptors of Broadly Neutralizing Antibody Lineages. mBio (2019) 10:1–18. doi: 10.1128/mBio.02810-18 PMC6391921 30808695

[53] Boyoglu-Barnum S Hutchinson G Boyington J Moin S Gillespie R Tsybovsky Y. Glycan Repositioning of Influenza Hemagglutinin Stem Facilitates the Elicitation of Protective Cross-Group Antibody Responses. Nat Commun (2020) 11:791. doi: 10.1038/s41467-020-14579-4 32034141 PMC7005838

[54] Garg A Mittal S Padmanabhan P Desikan R Dixit N. Increased B Cell Selection Stringency In Germinal Centers Can Explain Improved COVID-19 Vaccine Efficacies With Low Dose Prime or Delayed Boost. Front Immunol (2021) 12:776933. doi: 10.3389/fimmu.2021.776933 34917089 PMC8669483

[55] Galimidi R Klein J Politzer M Bai S Seaman M Nussenzweig M. Intra-Spike Crosslinking Overcomes Antibody Evasion by HIV-1. Cell (2015) 160:433–46. doi: 10.1016/j.cell.2015.01.016 PMC4401576 25635457

[56] Keating R Johnson J Brice D Labombarde J Dent A McGargill M. Broadly Reactive Influenza Antibodies Are Not Limited by Germinal Center Competition With High-Affinity Antibodies. mBio (2020) 11:1–13. doi: 10.1128/mBio.01859-20 PMC7642676 33144374

[57] Barnes C West A Huey-Tubman K Hoffmann M Sharaf N Hoffman P. Structures of Human Anti894 Bodies Bound to SARS-CoV-2 Spike Reveal Common Epitopes and Recurrent Features of Antibodies. Cell (2020) 182:828–42. doi: 10.1016/j.cell.2020.06.025 PMC7311918 32645326

[58] Robert P Arulraj T Meyer-Hermann M. Ymir: A 3d Structural Affinity Model for Multi-Epitope Vaccine Simulations. iScience (2021) 24:102979. doi: 10.1016/j.isci.2021.102979 34485861 PMC8405928

[59] Conti S Kaczorowski K Song G Porter K Andrabi R Burton D. Design of Immunogens to Nelicit Broadly Neutralizing Antibodies Against HIV Targeting the CD4 Binding Site. Proc Natl Acad Sci USA (2021) 118:1–12. doi: 10.1073/pnas.2018338118 PMC7936365 33637649

[60] Sprenger K Conti S Ovchinnikov V Chakraborty A Karplus M. Multiscale Affinity Maturation Simulations to Elicit Broadly Neutralizing Antibodies Against Hiv. PloS Comput Biol (2021). doi: 10.1101/2021.09.01.458482 PMC9020693 35442968

[61] Cohen A Yang Z Gnanapragasam P Ou S Dam K Wang H. Construction, Characterization, and Immunization of Nanoparticles That Display a Diverse Array of Influenza HA Trimers. PloS One (2021) 16:e0247963. doi: 10.1371/journal.pone.0247963 33661993 PMC7932532

[62] Casten L Pierce S. Receptor-Mediated B Cell Antigen Processing. Increased Antigenicity of a Globular Protein Covalently Coupled to Antibodies Specific for B Cell Surface Structures. J Immunol (Baltimore Md 1950) (1988) 140:404–10. 2447176

[63] Alberts B Johnson A Lewis J Raf M Roberts K Walter P. Molecular Biology of the Cell. 4th ed. New York: Garland Science (2002).

[64] Rundell A DeCarlo R HogenEsch H Doerschuk P. The Humoral Immune Response to Haemophilus Influenzae Type B: A Mathematical Model Based on T-Zone and Germinal Center B-Cell Dynamics. J Theor Biol (1998) 194:341–81. doi: 10.1006/jtbi.1998.0751 9778443

[65] Leanderson T Källberg E Gray D. Expansion, Selection and Mutation of Antigen-Specific B Cells in Germinal Centers. Immunol Rev (1992) 126:47–61. doi: 10.1111/j.1600-065x.1992.tb00630.x 1375924

[66] Press W Flannery B Teukolsky S Vetterling W. Numerical Recipes in FORTRAN 77: The Art of Scientific Computing. 2nd ed. Cambridge, UK: Cambridge University Press (1992).

[67] Anderson S Khalil A Uduman M Hershberg U Louzoun Y Haberman A. Taking Advantage: High-Affinity B Cells in the Germinal Center Have Lower Death Rates, But Similar Rates of Division, Compared to Low-Affinity Cells. J Immunol (Baltimore Md 1950) (2009) 183:7314–25. doi: 10.4049/jimmunol.0902452 PMC4106706 19917681

[68] Gitlin A Mayer C Oliveira T Shulman Z Jones M Koren A. HUMORAL IMMUNITY. T Cell Help Controls the Speed of the Cell Cycle in Germinal Center B Cells. Sci (New York NY) (2015) 349:643–6. doi: 10.1126/science.aac4919 PMC4809261 26184917

[69] Amitai A Mesin L Victora G Kardar M Chakraborty A. Population Dynamics Model for Clonal Diversity in a Germinal Center. Front Microbiol (2017) 8:1693. doi: 10.3389/fmicb.2017.01693 28955307 PMC5600966

[70] Hill TL. An Introduction to Statistical Thermodynamics. New York: Dover (1986).

[71] El Shikh M El Sayed R Sukumar S Szakal A Tew J. Activation of B Cells by Antigens on Follicular Dendritic Cells. Trends Immunol (2010) 31:205–11. doi: 10.1016/j.it.2010.03.002 PMC2886728 20418164

[72] Saphire E Parren P Pantophlet R Zwick M Morris G Rudd P. Crystal Structure of a Neutralizing Human IGG Against HIV-1: A Template for Vaccine Design. Sci (New York NY) (2001) 293:1155–9. doi: 10.1126/science.1061692 11498595

[73] Ise W Kurosaki T. Plasma Cell Differentiation During the Germinal Center Reaction. Immunol Rev (2019) 288:64–74. doi: 10.1111/imr.12751 30874351

[74] Kelsoe G. In Situ Studies of the Germinal Center Reaction. Adv Immunol (1995) 60:267–88. doi: 10.1016/S0065-2776(08)60587-8 8607371

[75] Murphy K. Janeway’s Immunobiology. eighth. New York: Garland Science (2012).

[76] Liu Y Johnson G Gordon J MacLennan I. Germinal Centres in T-Cell-Dependent Antibody Responses. Immunol Today (1992) 13:17–21. doi: 10.1016/0167-5699(92)90199-H 1739427

[77] Eaton JW Bateman D Hauberg S Wehbring R. GNU Octave Version 4.0.0 Manual: A High-Level Interactive Language for Numerical Computations. (2015).

[78] Moin P. Fundamentals of Engineering Numerical Analysis. Cambridge, UK: Cambridge University Press (2001).

