# Supplementary material for: A Coarse-Grained Model of Affinity Maturation Indicates the Importance of B-Cell Receptor Avidity in Epitope Subdominance
Source: Front Immunol. 2022 Mar 18;13:816634. doi: 10.3389/fimmu.2022.816634 (PMC8971376; doi:10.3389/fimmu.2022.816634)
Supplement: Supplementary file 1 [file DataSheet_1.pdf]

Supplementary Material

A coarse-grained model of affinity maturation indicates the importance of B-cell receptor avidity in epitope subdominance

V. Ovchinnikov[1,a] and M. Karplus[1,2,b]
[1] Harvard University, Department of Chemistry and Chemical Biology, Cambridge, MA, 02138, USA
[2] Laboratoire de Chimie Biophysique, ISIS, Université de Strasbourg, 67000 Strasbourg, France
[a] ovchinn@fas.harvard.edu; ovchinnv@georgetown.edu
[b] marci@tammy.harvard.edu

SUPPLEMENTARY TEXT AND FIGURES

The computer code to reproduce the simulation data and figures in this paper can be found online at `https://github.com/ovchinnv/affinity-maturation-paper2021.git`

S1 GC simulations with a variable ΔK_{eq} BCR#1 advantage

Because the affinity advantage of a BCR targeting a conserved epitope cannot be predicted accurately if antigenic drift is present, instead of modeling an affinity advantage of a particular BCR using a constant ΔK_{eq}, as done in the main text (see Fig. 6), a more realistic scenario is to treat ΔK_{eq} as a random variable. To this end, in the simulations described here we sample $\log_{10} \Delta K_{eq}$ from the Gaussian distribution centered on 2 (which corresponds to ΔK_{eq}=100) with variance 0.5 ; equivalently, ΔK_{eq} is sampled from the lognormal distribution. Each set of simulations is repeated 50 times to generate statistics. The distribution of ΔK_{eq} is shown in Fig. S4A (inset).

Figure S4 panels A and B show the MBC#1 fraction from the simulations of the bivalent and monovalent cases, respectively. The differences between the two cases are similar to those in previous simulations with fixed ΔK_{eq}. For the bivalent case, the average MBC#1 fraction for the competing cases $o \geq 0.9$, is around 30%–40% for 9 or more distracting epitopes, whereas for the monovalent case, this number is on the average about 3%. More importantly, in the bivalent case, some MBC#1 cells are always present, while in some individual simulations of the monovalent case, MBC#1 proportion is near zero (Fig. S4B).

S2 Solvent-accessible surface area of HA

Solvent-accessible surface areas (SASA) of the HA head, stem, and base regions were computed using the structure of H1N1 virus California/04/2009 from PDB entry 3LZG.Xu et al. (2010) The program CHARMMBrooks et al. (2009) with standard parametersBest et al. (2012) and water probe radius of 1.4Å was used for the SASA computation. The HA was partitioned into three regions, head, stem, and base, as shown in Fig. S5 in red, green, and blue, respectively. The corresponding SASA values were computed to be 314, 175, and 50 nm^2.

S3 GC simulations of the effect of AG#1 concentration with multiple competing BCRs

In Fig. 7 of the main text, it was shown that the antigen concentration has a large effect on B-cell and memory cell growth, as well as on the affinity of the corresponding BCRs. For brevity, we only showed the results of a single calculation with 3 BCR/epitope pairs with occlusion o=1. In this section, we repeat the calculations while systematically varying the number of BCR/epitope pairs (2–15), occlusion parameter (0,

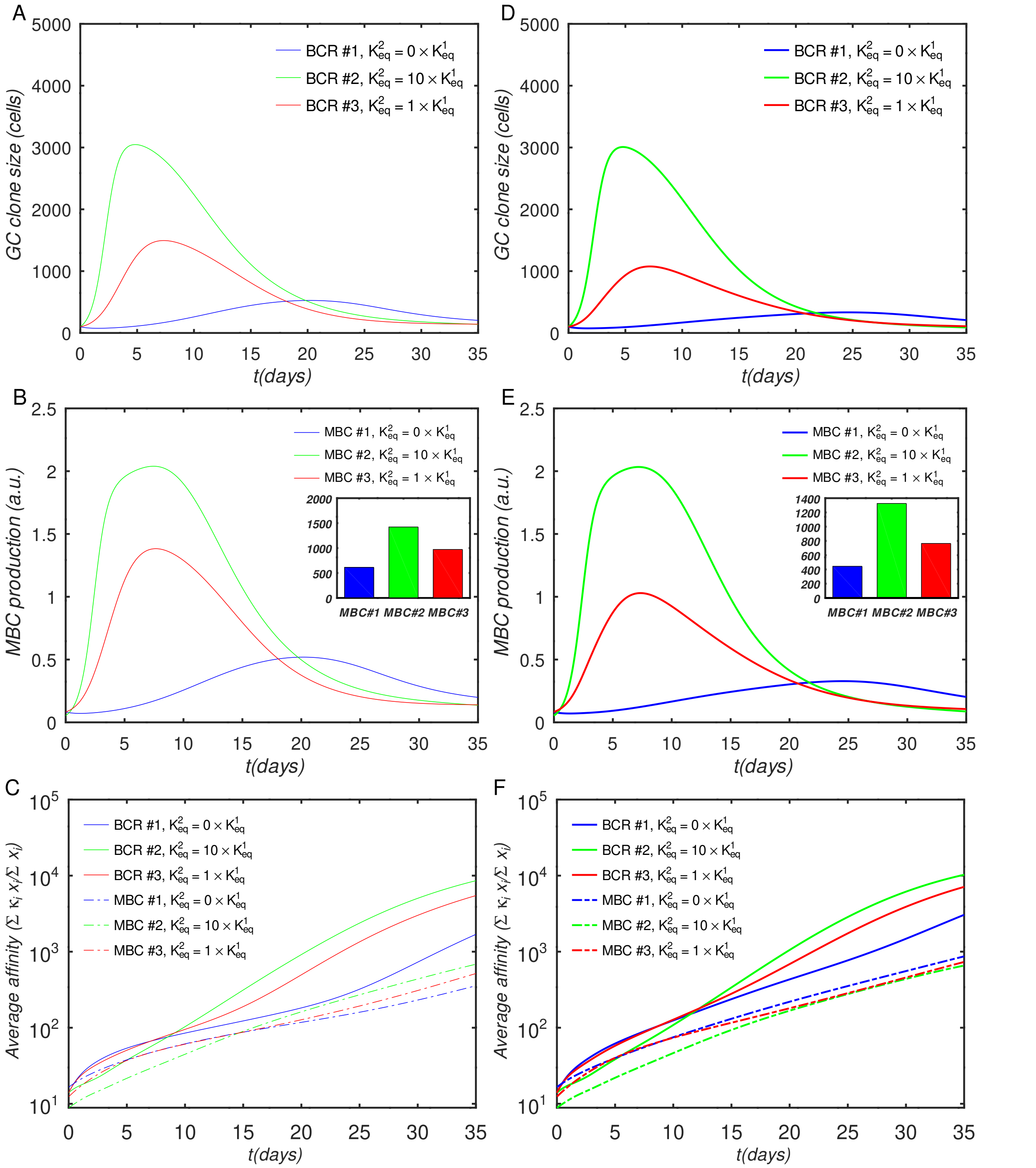

Figure S1. Effect of antibody valency and epitope occlusion on GC properties; different B-cell clones interact in the intermediate regime, *i.e.* left column (A–C): $o = 0.5$; Right column (D–F): $o = 0.9$; A,D: Total B cells; B,E: Memory cell production rate; insets: total MBC population at end of simulation; C,F: Average affinity of B-cells and MBCs.

0.5, 0.9, 1), affinity advantage provided to the BCR#1s ($\times 10$, $\times 100$, and $\times 1000$), and whether BCR#1s bound monovalently (K_{eq}^{12}=0) or bivalently (K_{eq}^{12}=10K_{eq}^{11}). The results are summarized in Fig. S7, after six consecutive GC simulations, with the relative MBC#1 output shown in figures Fig. S7A,B and average

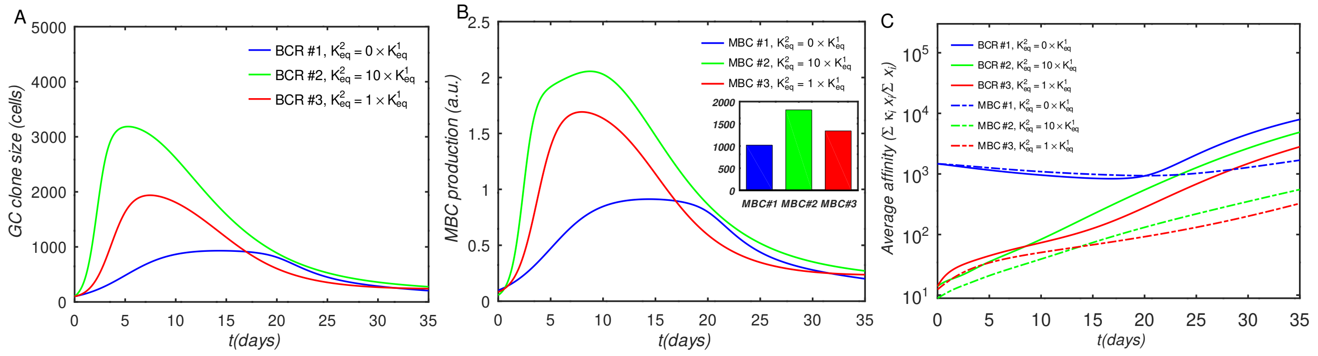

Figure S2. Effect of initial affinity advantage on the growth of monovalent B-cells in the independent (noninteracting) B-cell case ($o = 0$). Panels A–C show the same quantities as Fig. 5A–C; The affinity distribution corresponding to BCR#1 was shifted toward higher values relative to BCR#2 and BCR#3 (Fig. 5D, main text).

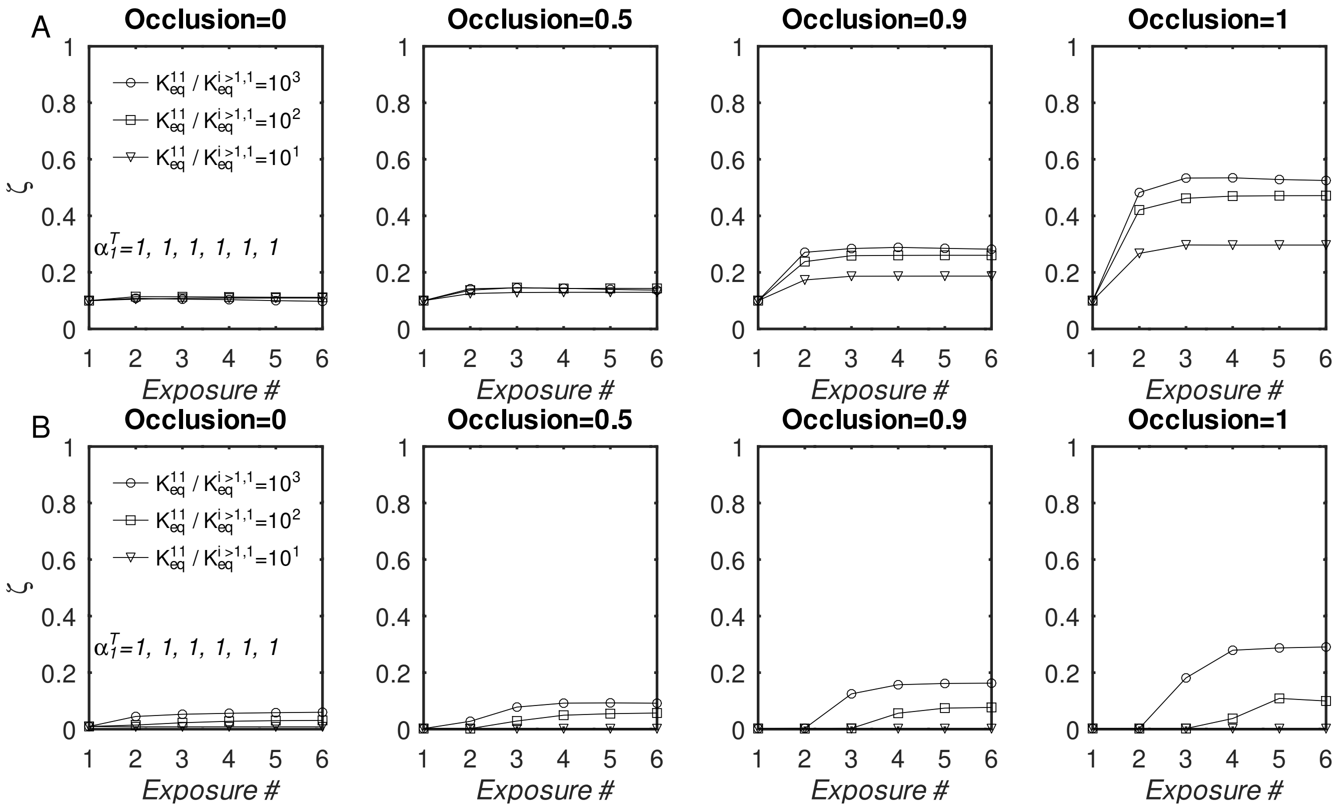

Figure S3. Fraction of MBC#1 *vs.* number of sequential GC simulations for different initial affinity advantage values with 10 total BCR/Epitope pairs. A: BCR#1 is cooperatively bivalent (K_{eq}^{12}=10K_{eq}^{11}); B: BCR#1 is monovalent (K_{eq}^{12}=0).

MBC#1 affinity, relative to that of the other MBCs, in Fig. S7C,D. The average affinity of MBC lineage i is computed as $\langle\kappa^i\rangle = \sum_{j=1}^{N_\epsilon} \kappa_j^i w_j^i / \sum_{j=1}^{N_\epsilon} w_j^i$, where w_j^i and κ_j^i are the nondimensional MBC concentrations and affinities, respectively (see Methods). For comparison, we also performed simulations with lower Ag#1 amounts, *i.e.* α_1^T=1 and α_1^T=1.5 in dimensionless units.

Figure S7 shows that increasing Ag concentration leads to a greater MBC output in all cases, with the increase being larger if the corresponding BCR also has a significant affinity advantage (shown for

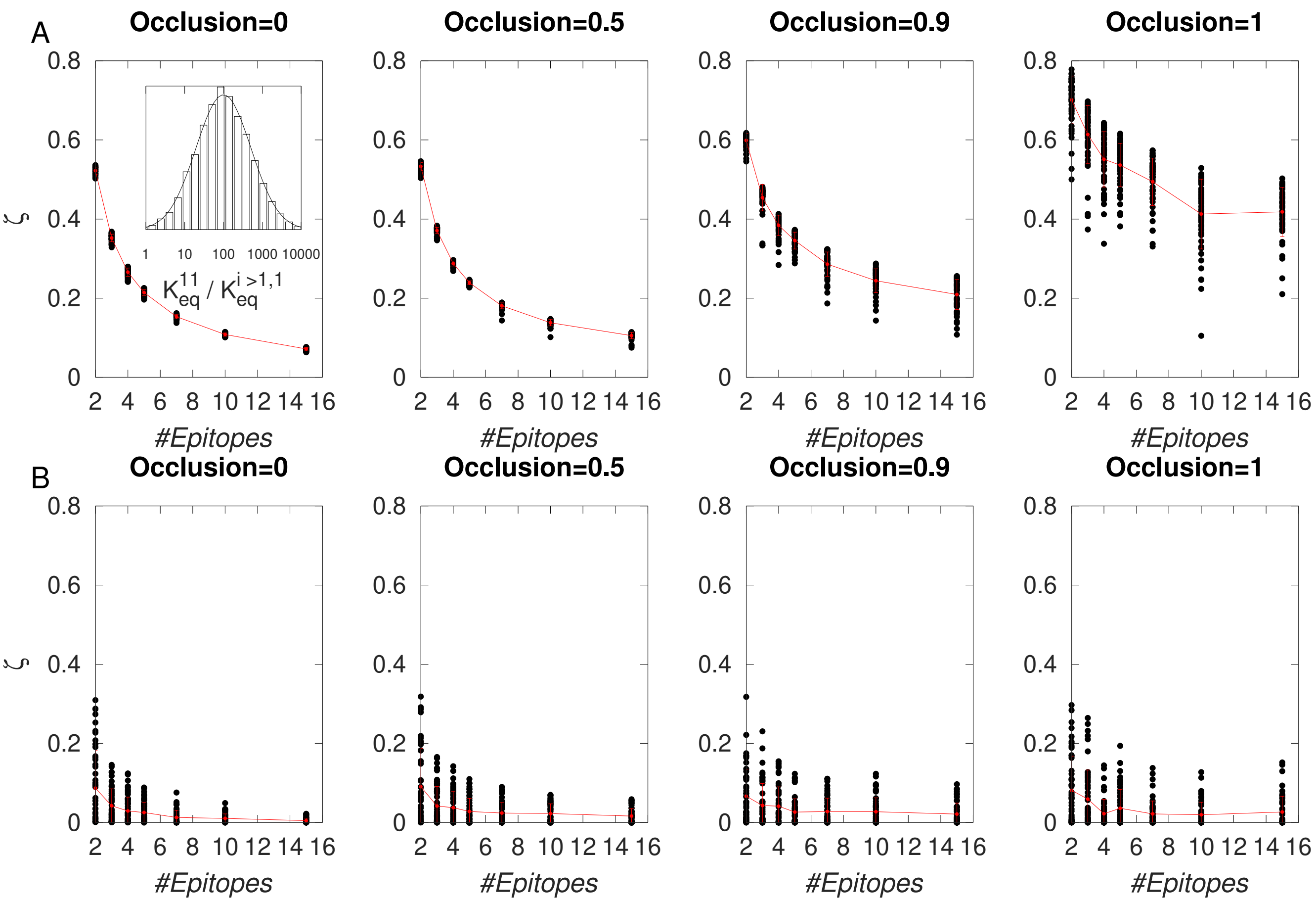

Figure S4. Fraction of MBC#1 at the end of six GC simulations for initial affinity advantage values drawn from the lognormal distribution (inset of panel A) *vs.* total number of BCR/Epitope pairs. A: BCR#1 is cooperatively bivalent (K_{eq}^{12}=10K_{eq}^{11}); B: BCR#1 is monovalent (K_{eq}^{12}=0). Red vertical bars represent standard deviation.

bivalent and monovalent BCR#1s in Figure S7A and Figure S7B, respectively). The increased MBC output corresponds to decreased affinity, however, the affinity decrease being larger for monovalent (Figure S7D), rather than for bivalent BCR#1s (Figure S7C). The simulation results therefore suggest that epitope subdominance can be overcome by increasing epitope concentration in vaccinations with cocktails of designed antigens, as proposed recently by others,Kanekiyo et al. (2019); Cohen et al. (2021); Ives et al. (2020) but at the expense of a reduction in the affinity of the resulting MBCs. For the practical purpose of vaccine design, the precise Ag concentrations may need to be optimized to achieve a compromise between MBC population size and affinity for antigen.

S4 Optimization of model parameters

To optimize the fit of the model predictions to the experimental data,Wittenbrink et al. (2011); Weisel et al. (2016) we used steepest (gradient) descent to minimize the squared error (SE) between the simulated and experimental GC size, MBC and PC production. To achieve similar weighting of the errors from the GC, MBC and PC comparisons, each of the three SE components was normalized by the corresponding mean-squared experimental value divided by the corresponding average experimental error bar.

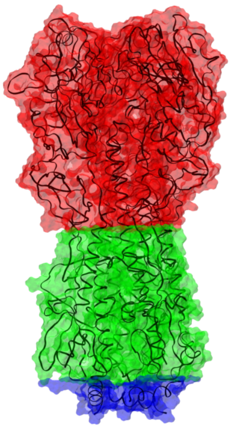

Figure S5. Illustration of a H1N1 hemagglutinin trimeric spike partitioned into the head (red, top), stem (green, middle), and base (blue, bottom) regions. The structure is taken from PDB entry 3LZGXu et al. (2010).

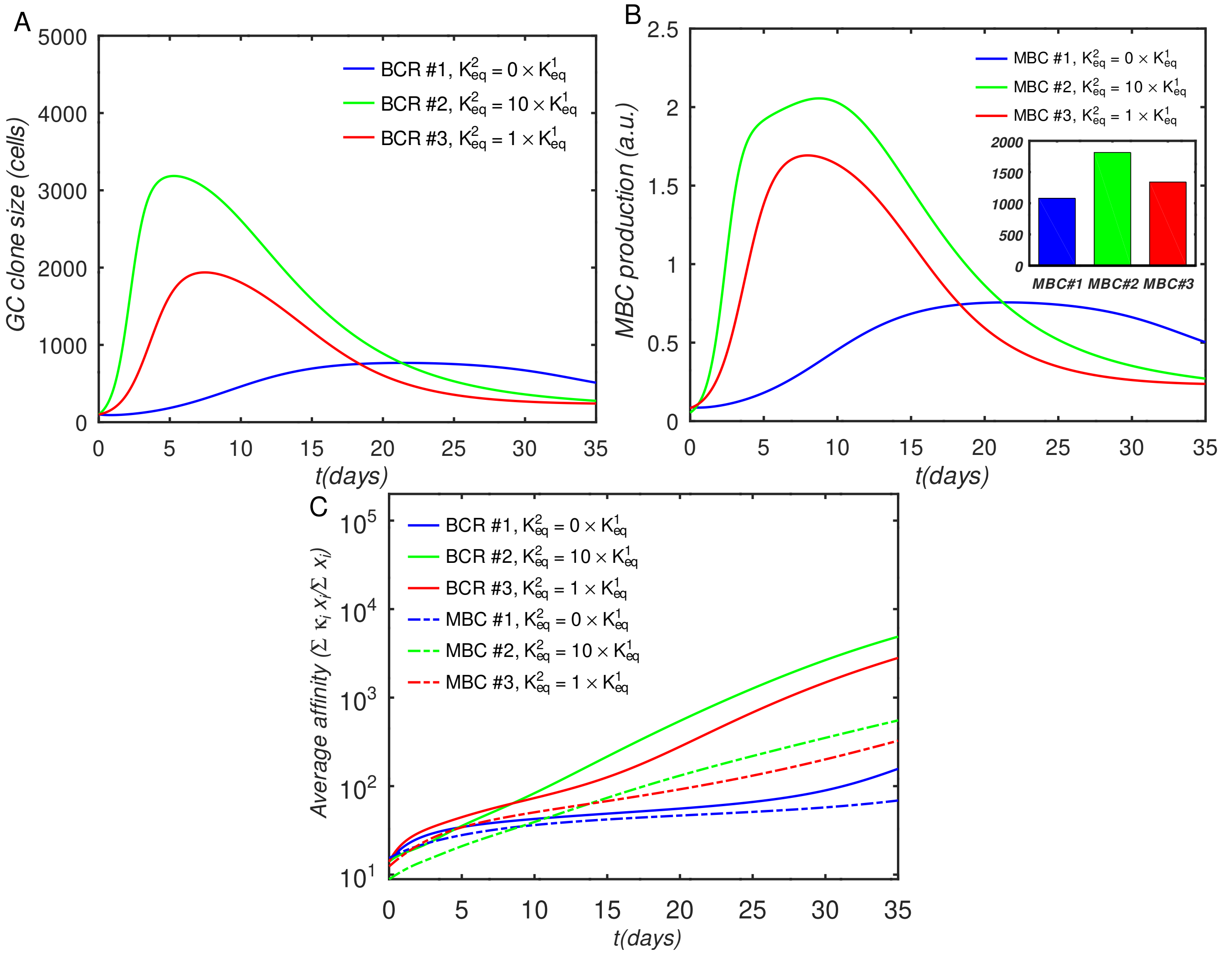

Figure S6. Effect of BCR valency and epitope concentration on GC evolution in the noninteracting regime (o=0); $\alpha_1^T = 2$, $\alpha_2^T = 1$, $\alpha_3^T = 1$; α^T is the nondimensional total Ag concentration. Results for the fully interacting case are shown in Fig. 7 (main text).

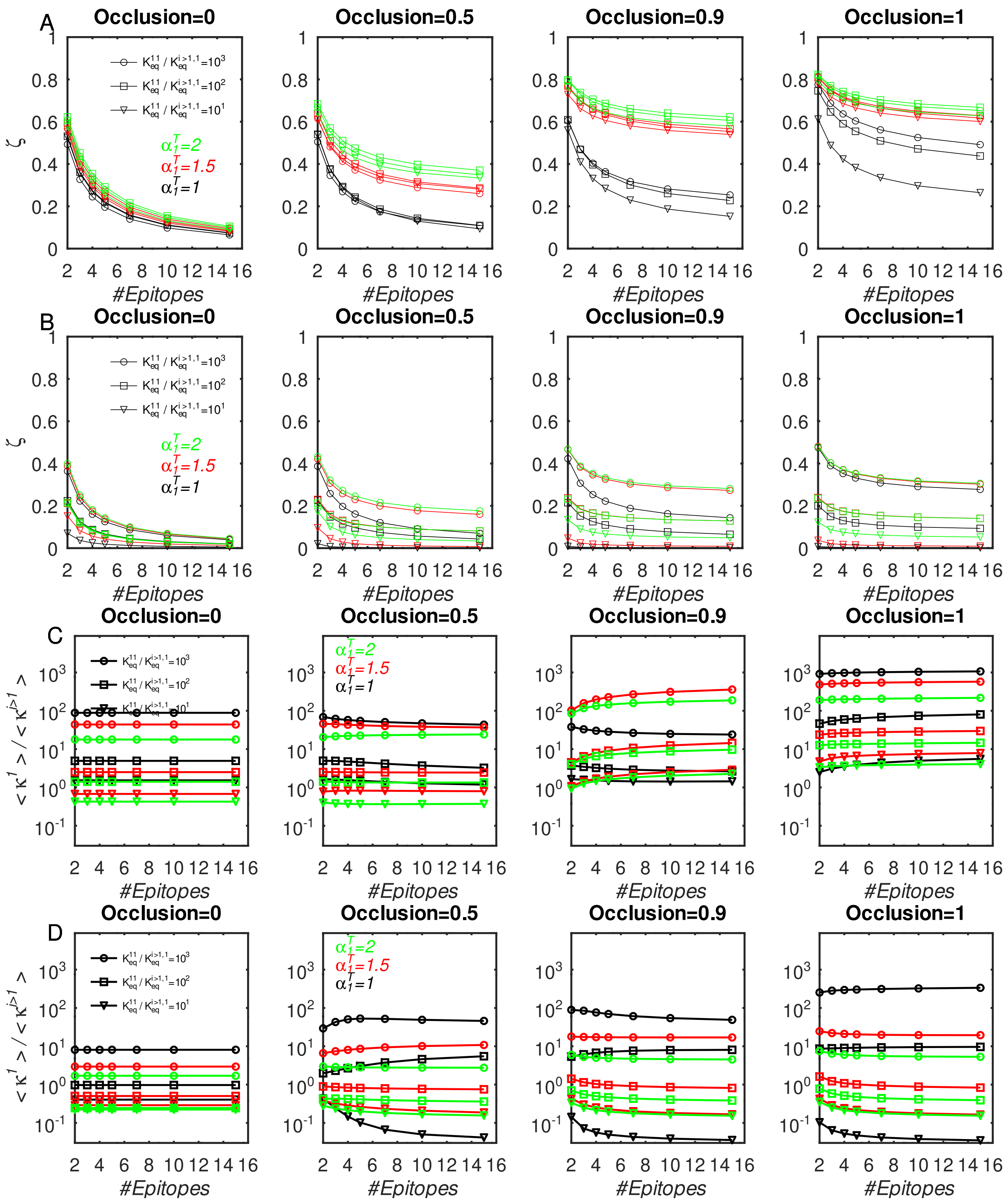

Figure S7. Results of six consecutive GC simulations with different initial affinity advantage values and epitope concentrations *vs.* total number of BCR/Epitope pairs. A & B: MBC#1 fraction (ζ) at the end of simulations. C & D: Ratio of average MBC#1 affinity to that of the other MBCs (see text); in A & C: BCR#1 is cooperatively bivalent (K_{eq}^{12}=10K_{eq}^{11}); in B & D: BCR#1 is monovalent (K_{eq}^{12}=0).

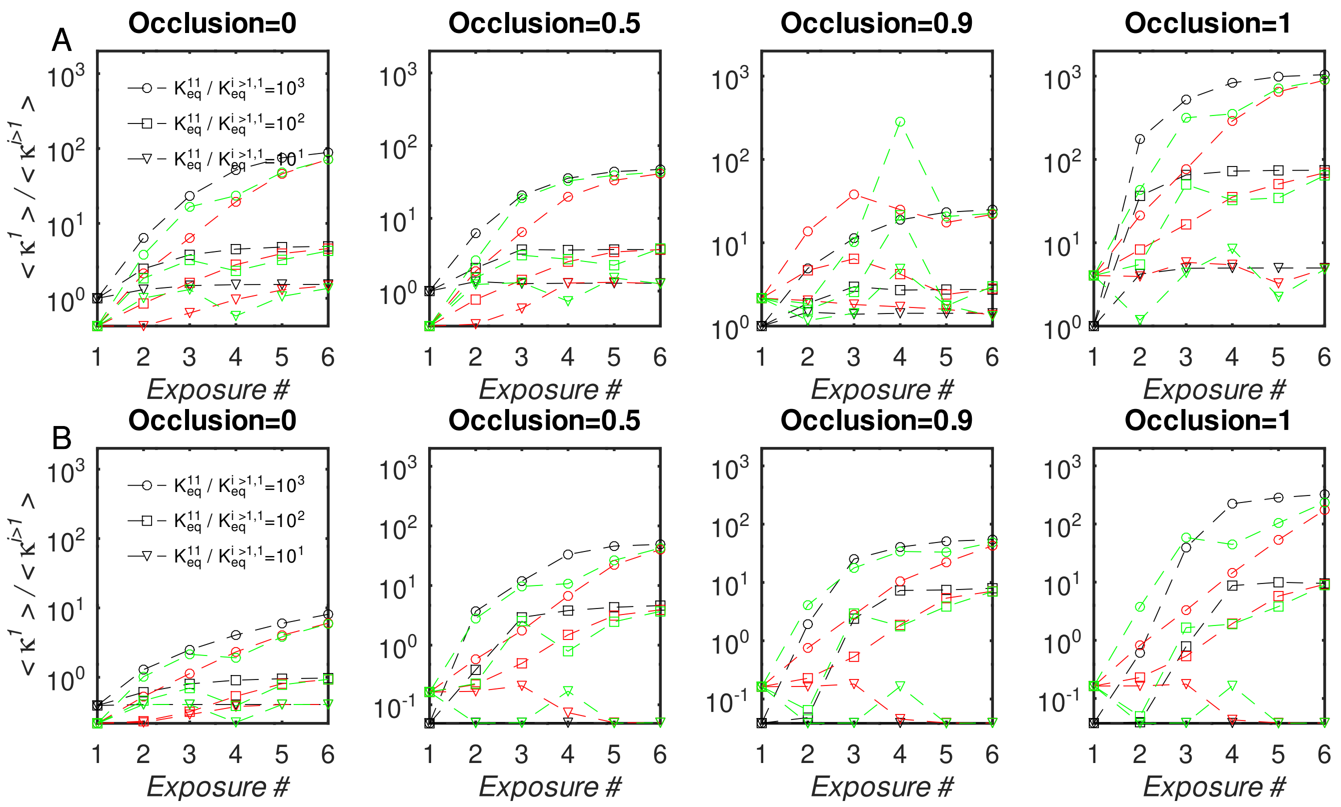

Figure S8. Affinity of BCR#1 *vs.* number of sequential GC simulations for different initial affinity advantage values and different AG#1 concentrations, with 10 total BCR/Epitope pairs. A & B: Ratio of average MBC#1 affinity to that of the other MBCs (see text); A: BCR#1 is cooperatively bivalent (K_{eq}^{12}=10K_{eq}^{11}); B: BCR#1 is monovalent (K_{eq}^{12}=0). The different colors correspond to different concentration profiles of Ag#1, as given in the main text, and in Fig. 8A.

The gradients of the SE with respect to a parameter, *e.g.* ϕ_i, were obtained using the second-order finite difference formula,*i.e.*

$$\frac{\partial SE^n}{\partial \phi_i} \simeq \frac{SE(\phi_i^n + \delta\phi_i) - SE(\phi_i^n - \delta\phi_i)}{2\delta\phi_i}, \tag{S1}$$

where ϕ_i^n is the value of the parameter at optimization iteration n, and $\delta\phi_i$ was the finite difference step set to 0.01. Parameter values were updated using

$$\phi_i^{n+1} = \phi_i^n - \Delta \times \frac{\partial SE^n}{\partial \phi_i}\,, \tag{S2}$$

where $\Delta \simeq 0.02$ was an empirically determined step size chosen small enough to reduce instabilities, but large enough to achieve relatively fast optimization, *e.g.* 100 steps computed within ~30 minutes of computer time.

The optimization described above is not very sophisticated, *e.g.*, conjugate gradient or Newton-Raphson minimization could be used to locate optimal parameter values with greater accuracy and speed; however, the simple steepest descent used here is sufficient for the present purposes, because (1) the accuracy of the fit need only be within experimental uncertainty, and (2) the optimization only needs to be performed once, and therefore its speed of convergence is not very important.

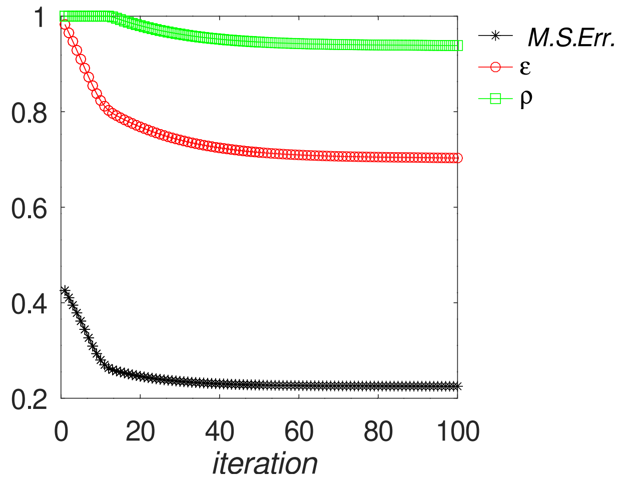

Figure S9. Illustration of parameter fitting using steepest descent minimization (see text).

In Fig. S9 we show the convergence of the parameters ϵ and ρ in the activation function Eq. 10. The values for both parameters were initially set to 1, and were constrained to the interval [0.5,1] by setting any value that was outside the interval after an iteration, to the nearer boundary. The optimization converges to $\epsilon \simeq 0.7$ and $\rho \simeq 0.94$ within about 100 iterations.

S5 Activated proliferation model

In Sec. 4 of the main text, we noted that the affinity maturation (AM) model used in this work implements B-cell selection *via* rescue from apoptosis of B-cells with the highest affinity to their cognate epitope (*a.k.a.* death-limited selectionAnderson et al. (2009); Zhang and Shakhnovich (2010); Amitai et al. (2017)). However, there is evidence that B-cell activation actually increases the rate of proliferation of the corresponding B-cells in the dark zone.Gitlin et al. (2015)

Mathematically, the activated model differs from the non-activated model (Eq. 2) in that the proliferation rate constant is scaled by the activation function, *i.e.*,

$$\frac{d[B_j^i]}{dt} = \left\{-k_d(1-h_j^i) - k_p h_j^i\right\}[B_j^i] + 2k_p \sum_{k=1}^{N_\epsilon} m_{kj}[B_k^i]h_k^i, \quad \text{for } 1 \leq i \leq N_B. \tag{S3}$$

Because the modeling described here involves substantial fitting and parametrization, and because the experimental data has high uncertainties,Wittenbrink et al. (2011); Weisel et al. (2016) it was possible to fit the data within the experimental uncertainties with either model. In Fig. S10, we show the validation calculations performed with one Ag using the activated proliferation model. All of the simulation constants and parameters were kept as in Table 1, except for modifications to the proliferation and death rate constants that were needed to obtain a good fit to the experiment; specifically k_p^{max}=2.55/d, and k_d=2.25/d. In partcular, the sizeable decrease in k_d was needed to compensate for a decrease in the proliferation rate caused by scaling the rate constant k_p by the activation factor h_j^i, which is always below 1.

Compared with the results of the non-activated proliferation model in Fig. 3 (main text), the maximum B-cell population is higher, but remains well within the experimental dispersion.

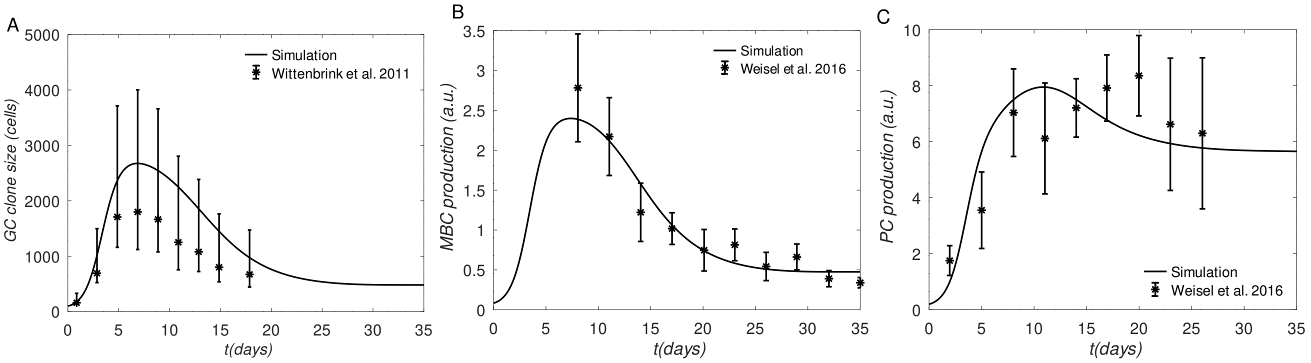

Figure S10. Comparison of experiments and simulations using the activated proliferation model (see text). A: Total B cells, B: Memory cell production rate, C: Plasma cell production rate. Experimental data for A was generated from the GC cross-sectional areas plotted in Fig. S1B of Ref. 7, and converted to B cell counts as done in Ref. 13; experimental data for B & C was taken from Fig. 4 of Ref. 13, who used raw data from Weisel et al. (2016).

Furthermore, we repeated several simulations with multiple BCR/Ag pairs to check that our conclusions remained qualitatively the same. For example, in Fig. S11 we show the results of simulating three BCR/Ag pairs evolving independently ($o = 0$, Fig. S11A–C), *vs.* evolving while fully interacting with each other ($o = 1$, Fig. S11D–F). Comparing the results with those derived from the non-activated model (Fig. 4 in the main text), it is clear that the two models are in agreement in terms of the relative ranking of the cells according to population size or affinity.

Thus the results suggest that the choice of activated *vs.* nonactivated proliferation is not likely to impact qualitatively the conclusions in this study.

S6 T-cell-dependent B-cell activation

A key component in the GC model presented here is the activation function h, which determines which B-cells are rescued from apoptosis (Eq. 2) or, in the case of activated proliferation, receive a proliferation advantage. In the simulations described in the main text, we assumed that h has a simple dependence on the fraction of bound BCR Fabs (see Eqs. (8) and (10)). In reality, the survival of B-cells also depends on the extent of their interactions with helper T-cells (Tfh),Liu et al. (1989); Allen et al. (2007); Crotty (2014) and thus it is desirable to have available a more realistic model of activation that treats these interactions. In this section, we describe a simple T-cell model related to that of Mayer et al. (2019), and use it to compute a T-cell-based function h_T, as a replacement for h in Eq. 10. We compare the outcomes of trial AM simulations performed with h *vs.* h_T, and conclude that the use of h_T would not change qualitatively the conclusions of this study. However, the model could be used as a starting point for future studies, *e.g.*, of the effects of changing T-cell concentration, or other properties that could impact Tfh and B-cell survival and proliferation.

We assume that each B-cell B^i_j has a constant total number of major histocompatibility complexes of type II ($MHC2^T$) which can be unloaded ($MHC2^{i0}_j$) or loaded with an Ag-derived peptide ($MHC2^{iL}_j$). The total concentration of MHC2's in the GC available for binding a T-cell receptor (TCR) is

$$[MHC2^T] = MHC2^T \times \sum_{j=1}^{N_\epsilon} \sum_{i=1}^{N_B} [B^i_j], \tag{S4}$$

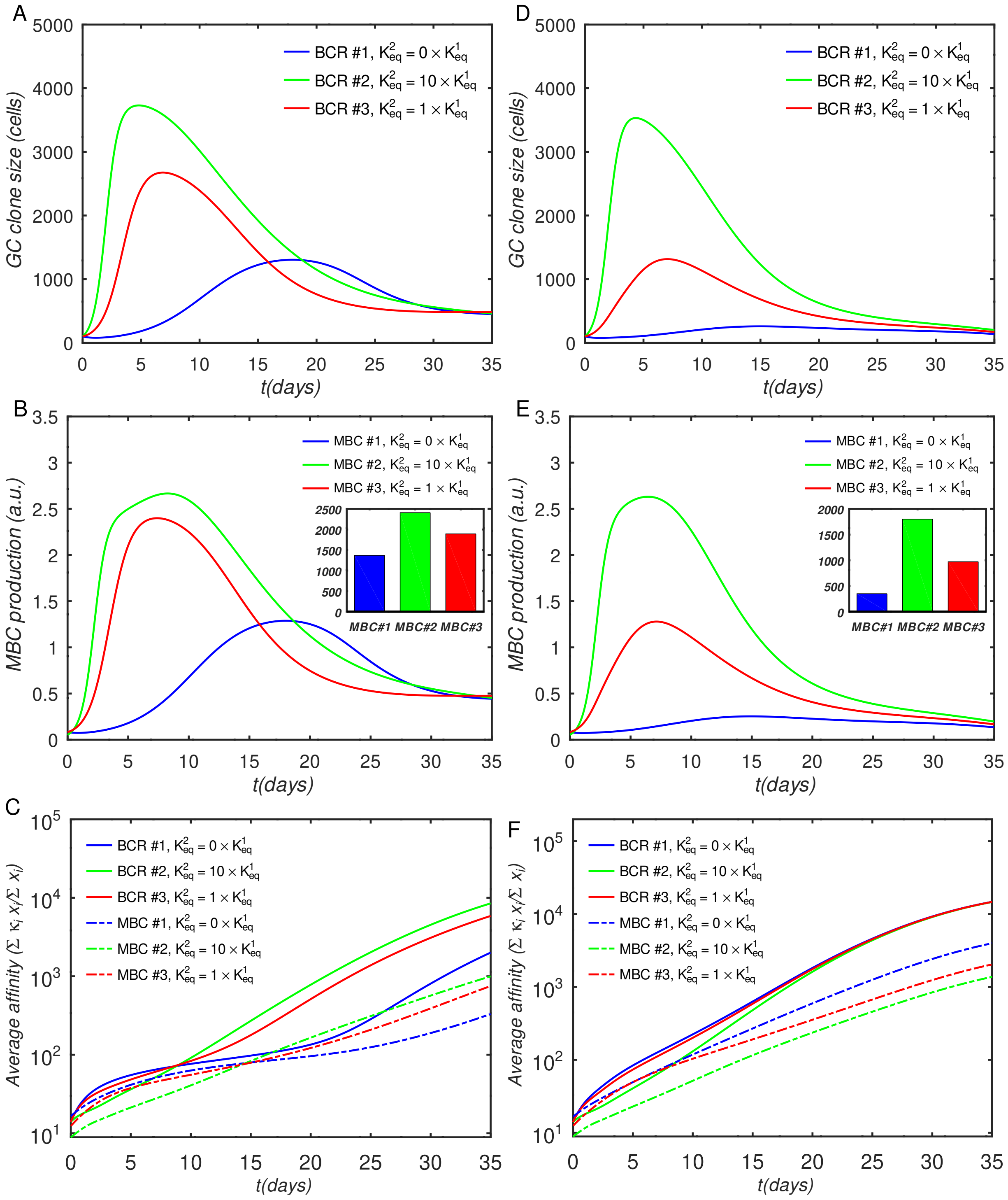

Figure S11. Effect of antibody valency and epitope occlusion on GC properties. Simulated using the activated proliferation model. Left column (A–C): noninteracting B-cell case ($o = 0$); Right column (D–F): fully interacting B-cell case ($o = 1$); A,D: Total B cells; B,E: Memory cell production rate; insets: total MBC population at end of simulation; C,F: Average affinity of B-cells and MBCs.

and the corresponding total loaded and unloaded MHC2 concentrations are

$$[MHC2^L] = \sum_{j=1}^{N_\epsilon} \sum_{i=1}^{N_B} [B_j^i] \times MHC2_j^{iL}, \tag{S5}$$

and

$$[MHC2^0] = [MHC2^T] - [MHC2^L], \tag{S6}$$

respectively.

We now postulate that unloaded and loaded MHC2's can bind to a TCR with equilibrium binding constants K_0^T and K_L^T, respectively. Given a total concentration of TCRs $[T^T]$, the unbound TCR concentration obeys a TCR conservation equation analogous to Eq. 9Kepler and Perelson (1993):

$$[T] + [MHC2^L]\theta_L^T + [MHC2^0]\theta_0^T = [T^T], \tag{S7}$$

where

$$\theta_L^T = \frac{K_L^T[T]}{1 + K_L^T[T]}, \tag{S8}$$

and

$$\theta_0^T = \frac{K_0^T[T]}{1 + K_0^T[T]} \tag{S9}$$

are the fractions of loaded and unloaded MHC2 receptors, respectively, that are bound to a TCR. Since we assume that there are only two types of MHC2 receptors (loaded and unloaded), Equation (S7) is equivalent to a quadratic polynomial in $[T]$, with the solutionMayer et al. (2019) given by the quadratic formula.

Solving Eq. (S7) requires the setting of several parameters. First, we specify $MHC2_j^{iL}$ and $MHC2_j^{i0}$ using the following simplifying assumptions: (1) the fraction of loaded MHC2's is equal to the the fraction of bound BCR arms, *i.e.*,

$$\frac{MHC2_j^{iL}}{MHC2^T} = \theta_j^i, \tag{S10}$$

and (2) we initially set the total number of MHC2 molecules displayed on the B-cell surface to the number of BCRs (*i.e.* σ in Table 1). However, this number had to be empirically reduced to 10^4 (10% of the number of BCRs) to match the experimental observation that the GC T-cell population is below ~20% of the total GC sizeAllen et al. (2007). We note that there does not seem to be a biological justification for this reduction, which may therefore indicate a deficiency in the model.

Second, we need to specify the MHC2/TCR binding constants K^T. To set K_L^T to a reasonable value, we assume that the maximum TCR-bound fraction of MHC2 at saturating antigen and a physiological helper T-cell concentration $[T^T]$ (to be specified below) is θ_{max}^T=0.9. In this regime, we approximate $[MHC2^0] \simeq 0$, and attribute the bound MHC2 fraction entirely to peptide-loaded MHC2, which implies

$$\theta_{max}^T = \theta_L^T. \tag{S11}$$

Solving Eq. (S11) for K_L^T and eliminating $[T]$ using Eq. (S7) gives

$$K_L^T = \frac{\theta_{max}^T}{([T^T] - \theta_{max}^T[MHC2^T])(1 - \theta_{max}^T)}. \tag{S12}$$

Similarly, we assume that when antigen is depleted, the TCR-bound fraction of MHC2, which in this case is attributable to unloaded MHC2, is $\theta^T_{min} = 0.01 = \theta^T_0$. Solving for K^T_0 yields

$$K^T_0 = \frac{\theta^T_{min}}{([T^T] - \theta^T_{min}[MHC2^T])(1 - \theta^T_{min})}. \tag{S13}$$

To compute the actual K^T values, we assume the total T-cell concentration $[T^T]$ to be 200 cells, or $\sim$10% of the average GC peak of Wittenbrink et al. (2011), which is roughly consistent with GC imaging.Allen et al. (2007) Further, we assume that maximal binding cannot occur when $[MHC2^T]$ exceeds $[T^T]$, so we can set $[MHC2^T] = [T^T]$ in Eqs. (S12) and (S13). With these assumptions,

$$K^T_L = \frac{\theta^T_{max}}{[T^T](1 - \theta^T_{max})^2} = 4.5 \times 10^{-1}/cell \tag{S14}$$

and

$$K^T_0 = \frac{\theta^T_{min}}{[T^T](1 - \theta^T_{min})^2} = 5.1 \times 10^{-5}/cell. \tag{S15}$$

For each B-cell, the TCR-bound fraction is given by

$$\theta^{iT}_j = \theta^T_L \theta^i_j + \theta^T_0 (1 - \theta^i_j), \tag{S16}$$

where the first and second terms on the right-hand side represent contributions from loaded and unloaded MHC2's, respectively. To use the T-cell-dependent B-cell activation model, we replace θ^i_j with θ^{iT}_j in Eq. 10.

We now consider the evolution of helper T-cells inside the GC. We assume that the T-cells whose receptors bind MHC2 complexes on B-cells undergo increased proliferation. Specifically,

$$\frac{d[T^T]}{dt} = k^T_p \left([T^T] - [T]\right) - k^T_d [T^T], \tag{S17}$$

where $k^T_p = 1.25/d$ and $k^T_d = 0.25/d$ are T-cell proliferation and death constants, respectively, which were manually adjusted from the T-cell model of Mayer et al. (2019), who used $k^T_p = 1.5/d$ and $k^T_d = 0.2/d$, to improve agreement with experimental data.

We note that the above model of the effects of T-cells on GC dynamics is heavily coarse grained, both spatially and temporally. For example, B-cells and T-cells interact preferentially in the light zone, and the duration, as well as the number of distinct encounters appear to be important.Allen et al. (2007) These behaviors are captured to some extent in more complex models,Meyer-Hermann et al. (2012); Pélissier et al. (2020) but not in ours. Further, only a single type of TCR is included here, which does not discriminate between the different peptide antigens that can be presented on MHC2s. However, increased model complexity would require additional experimental data for parametrization and validation. Because they are not available, we restrict ourselves to qualitative-level predictions in this study.

We first use the T-cell model to repeat the validation calculations of the single-AG case. Aside from the use of the T cell model, all simulation parameters were kept the same as in Table 1. In Fig. S12, we show the total B and T cells (panel A), MBC production (panel B) and PC production (panel C). Comparing

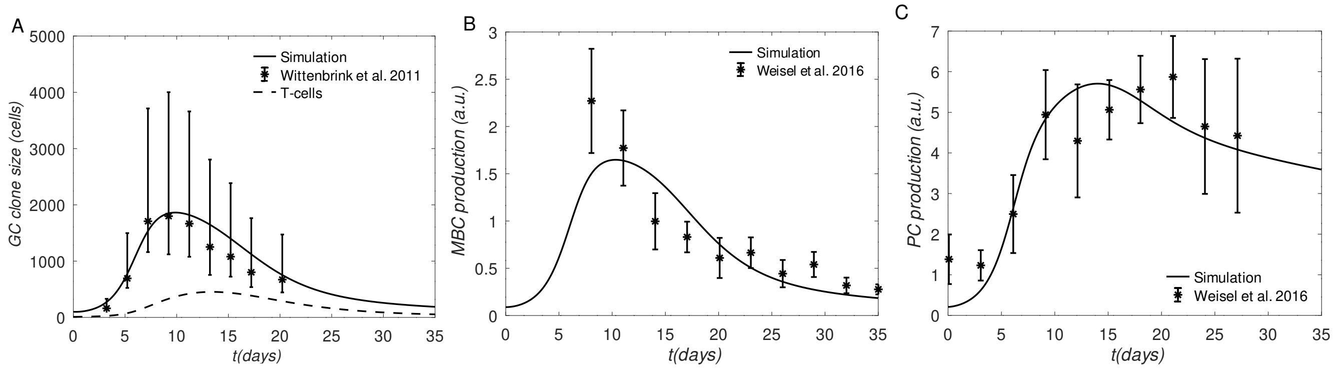

Figure S12. Comparison of experiments and simulations using the T-cell help model with non-activated proliferation (see text). A: B-cells and T-cells, B: Memory cell production rate, C: Plasma cell production rate.

with the results of the Ag-only activation model (see Fig. 3 in the main text), the main difference is a $\sim$2 day delay in the growth of all quantities. The reason for the delay is the extra time required for T-cell expansion, before sufficient B-cell activation can take place. The initial T-cell population was set to 10 cells, corresponding to 10% of the initial B-cell population. The peak T-cell population reached about 400 cells at 12 days, or 20% of the maximum B-cell population of $\sim$2000 cells, which is qualitatively consistent with known GC composition.Allen et al. (2007)

Next, in Fig. S13 we show the results of GC simulations of three BCR/Ag pairs, in the interacting and non-interacting cases, as was done in Fig. 4 (main text) for the Ag-activated model. Comparison of the two figures shows that the results are qualitatively the same. Therefore, the use of the present T-cell model throughout would not be expected to change the conclusions of this study. A possible explanation for the lack of significant differences between the Ag-activated and T-cell activated simulation results is that, in both cases, the models were first calibrated to match the same experimental data. Furthermore, the activation provided by the T-cell model ultimately comes from BCRs binding to Ags, *i.e.*, it is implicitly Ag-activated. While more complicated T-cell models would likely cause larger differences in the GC population predictions, our objective in this study was to only investigate the robustness of our predictions of the effects of BCR/Ag valency on B-cell competition with respect to changes to the GC model.

REFERENCES

Xu R, Ekiert D, Krause J, Hai R, Crowe J, Wilson I. Structural Basis of Preexisting Immunity to the 2009 H1N1 Pandemic Influenza Virus. *Science* **328** (2010) 357–60. doi:10.1126/science.1186430.

Brooks B, Brooks III C, Mackerell Jr A, Nilsson L, Petrella R, Roux B, et al. CHARMM: The Biomolecular Simulation Program. *J. Comput. Chem.* **30** (2009) 1545–1614. PMC2810661.

Best R, Zhu X, Shim J, Lopes P, Mittal J, Feig M, et al. Optimization of the Additive CHARMM All-Atom Protein Force Field Targeting Improved Sampling of the Backbone ϕ, ψ and Side-Chain χ_1 and χ_2 Dihedral Angles. *J. Chem. Theor. Comput.* **8** (2012) 3257–3273.

Kanekiyo M, Joyce M, Gillespie R, Gallagher J, Andrews S, Yassine H, et al. Mosaic Nanoparticle Display of Diverse Influenza Virus Hemagglutinins Elicits Broad B Cell Responses. *Nat. Immunol.* **20** (2019) 362–372. doi:10.1038/s41590-018-0305-x.

Cohen A, Gnanapragasam P, Lee Y, Hoffman P, Ou S, Kakutani L, et al. Mosaic Nanoparticles Elicit Cross-Reactive Immune Responses to Zoonotic Coronaviruses in Mice. *Science (New York, N.Y.)* **371** (2021) 735–741. doi:10.1126/science.abf6840.

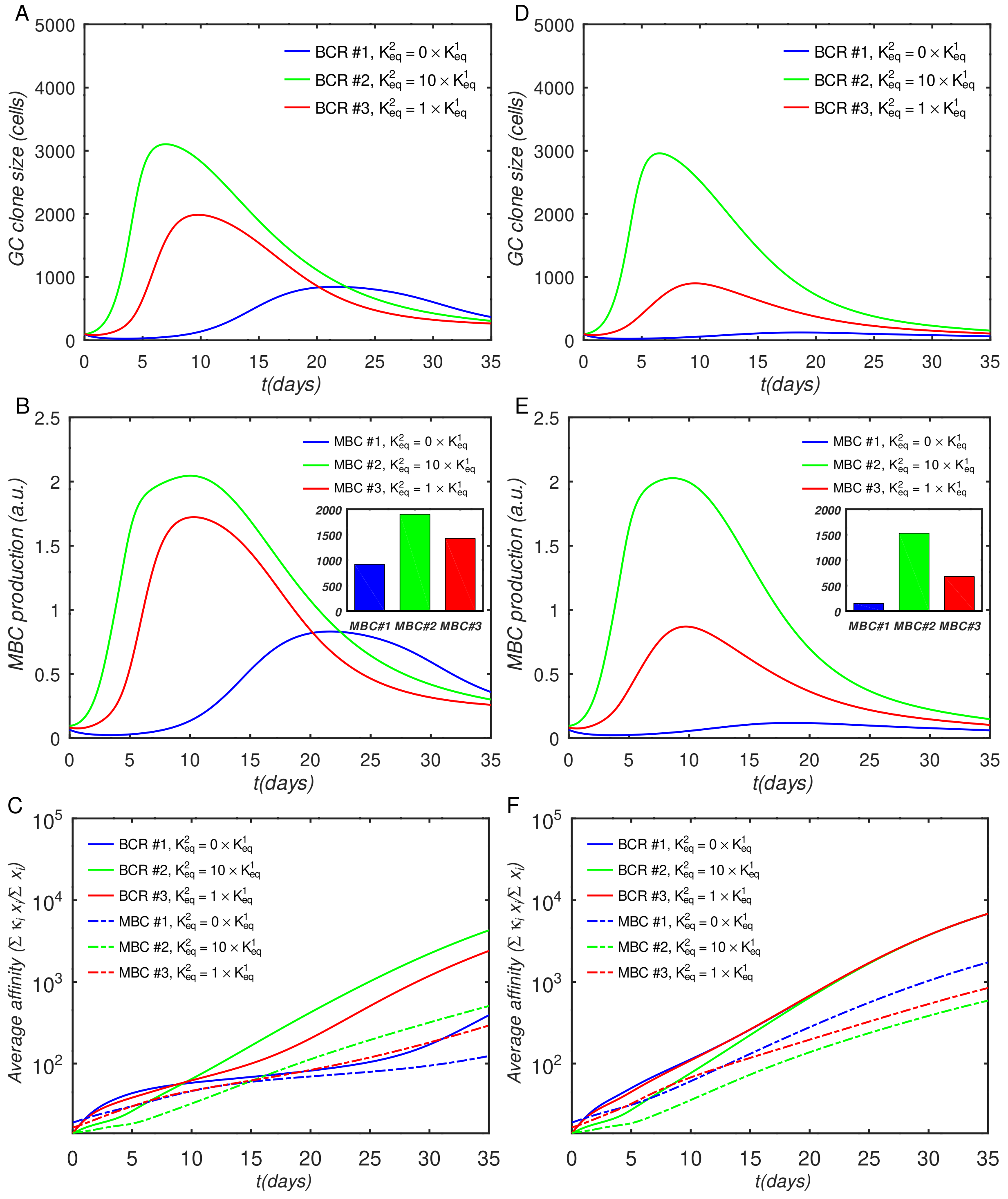

Figure S13. Effect of antibody valency and epitope occlusion on GC properties. Simulated using T-cell model with non-activated proliferation. Left column (A–C): noninteracting B-cell case ($o = 0$); Right column (D–F): fully interacting B-cell case ($o = 1$); A,D: Total B cells; B,E: Memory cell production rate; insets: total MBC population at end of simulation; C,F: Average affinity of B-cells and MBCs.

Wittenbrink N, Klein A, Weiser A, Schuchhardt J, Or-Guil M. Is There a Typical Germinal Center? a Large-Scale Immunohistological Study on the Cellular Composition of Germinal Centers During the Hapten-Carrier-Driven Primary Immune Response in Mice. *J. Immunol. (Baltimore, Md. : 1950)* **187** (2011) 6185–96. doi:10.4049/jimmunol.1101440.

Weisel F, Zuccarino-Catania G, Chikina M, Shlomchik M. a Temporal Switch in the Germinal Center Determines Differential Output of Memory B and Plasma Cells. *Immunity* **44** (2016) 116–130. doi:10.1016/j.immuni.2015.12.004.

Anderson S, Khalil A, Uduman M, Hershberg U, Louzoun Y, Haberman A, et al. Taking Advantage: High-Affinity B Cells in the Germinal Center Have Lower Death Rates, but similar rates of division, compared to low-affinity cells. *J. Immunol. (Baltimore, Md. : 1950)* **183** (2009) 7314–25. doi:10.4049/jimmunol.0902452.

Zhang J, Shakhnovich E. Optimality of Mutation and Selection in Germinal Centers. *PLoS Comput. Biol.* **6** (2010) e1000800. doi:10.1371/journal.pcbi.1000800.

Amitai A, Mesin L, Victora G, Kardar M, Chakraborty A. a Population Dynamics Model for Clonal Diversity in a Germinal Center. *Front. Microbiol.* **8** (2017) 1693. doi:10.3389/fmicb.2017.01693.

Gitlin A, Mayer C, Oliveira T, Shulman Z, Jones M, Koren A, et al. HUMORAL IMMUNITY. T Cell Help Controls the Speed of the Cell Cycle in Germinal Center B Cells. *Science (New York, N.Y.)* **349** (2015) 643–6. doi:10.1126/science.aac4919.

Pélissier A, Akrout Y, Jahn K, Kuipers J, Klein U, Beerenwinkel N, et al. Computational Model Reveals a Stochastic Mechanism Behind Germinal Center Clonal Bursts. *Cells* **9** (2020) 0. doi:10.3390/cells9061448.

Liu Y, Joshua D, Williams G, Smith C, Gordon J, MacLennan I. Mechanism of Antigen-Driven Selection in Germinal Centres. *Nature* **342** (1989) 929–31. doi:10.1038/342929a0.

Allen C, Okada T, Cyster J. Germinal-Center Organization and Cellular Dynamics. *Immunity* **27** (2007) 190–202. doi:10.1016/j.immuni.2007.07.009.

Crotty S. T Follicular Helper Cell Differentiation, function, and roles in disease. *Immunity* **41** (2014) 529–42. doi:10.1016/j.immuni.2014.10.004.

Mayer A, Zhang Y, Perelson A, Wingreen N. Regulation of T Cell Expansion by Antigen Presentation Dynamics. *Proc. Natl. Acad. Sci. USA* **116** (2019) 5914–5919. doi:10.1073/pnas.1812800116.

Kepler T, Perelson A. Somatic Hypermutation in B Cells: An Optimal Control Treatment. *J. Theor. Biol.* **164** (1993) 37–64. doi:10.1006/jtbi.1993.1139.

Meyer-Hermann M, Mohr E, Pelletier N, Zhang Y, Victora G, Toellner K. a Theory of Germinal Center B Cell Selection, division, and exit. *Cell Rep.* **2** (2012) 162–74. doi:10.1016/j.celrep.2012.05.010.
